# The E3 ubiquitin ligase Trim13 regulates Nur77 stability *via* casein kinase 2α

**DOI:** 10.1038/s41598-018-32391-5

**Published:** 2018-09-17

**Authors:** Bin Huang, Han Zhong Pei, Hyeun-Wook Chang, Suk-Hwan Baek

**Affiliations:** 1 Department of Biochemistry & Molecular Biology, College of Medicine, Yeungnam University, Daegu, South Korea; 2 College of Pharmacy, Yeungnam University, Gyeongsan, South Korea

## Abstract

Nur77 is a member of the NR4A subfamily of nuclear receptors and has been shown to regulate various biological processes such as apoptosis and inflammation. Here, we show that Nur77 ubiquitination is mediated by the tripartite motif 13 (Trim13), a RING-type E3 ubiquitin ligase. The interaction between Nur77 and Trim13 was confirmed by co-immunoprecipitation. Moreover, we found that Lys539 in Nur77 ubiquitination is targeted for Trim13, which leads to Nur77 degradation. The Trim13-mediated ubiquitination of Nur77 was optimal in the presence of the E2 enzyme UbcH5. Importantly, in addition to Trim13-mediated ubiquitination, the stability of Nur77 was also regulated by casein kinase 2α (CK2α). Pharmacological inhibition of CK2 markedly increased Nur77 levels, whereas overexpression of CK2α, but not its inactive mutant, dramatically decreased Nur77 levels by promoting Nur77 ubiquitination. CK2α phosphorylated Ser154 in Nur77 and thereby regulated Nur77 protein levels by promoting its ubiquitin-mediated degradation. Importantly, we also show that degradation of Nur77 is involved in TNFα-mediated IL-6 production *via* CK2α and Trim13. Taken together, these results suggest that the sequential phosphorylation and ubiquitination of Nur77 controls its degradation, and provide a therapeutic approach for regulating Nur77 activity through the CK2α-Trim13 axis as a mechanism to control the inflammatory response.

## Introduction

Nur77, also known as NGF1B (nerve growth factor 1B), TR3, or NR4A1 (nuclear receptor subfamily 4 group member 1), was the first member of the NR4A family to be identified as a gene induced by NGF in PC12 cells^1^. Nur77 is an immediate early response gene and plays a variety role in response to diverse stimuli including cytokines, stress, and apoptotic signals^2–4^. Nur77 has been implicated in many pathological conditions, including cancer, immune disorders, and metabolic or neurological diseases^5^. It has been reported to have a regulation of inflammatory responses. For example, *in vitro* studies showed a rapid induction of Nur77 expression by lipopolysaccharide (LPS) in macrophages^6^, and overexpression of Nur77 resulted in attenuation of cytokines and chemokines^7,8^. *In vivo*, Nur77 knock-out mice are more susceptible to a diseases including sepsis^9^, atherosclerosis^10^, airway inflammatory disease^7^. However, unlike other nuclear receptors whose functions are modulated by their ligands, endogenous ligands for Nur77 have not yet been identified. Therefore, regulation of Nur77 levels has been the primary focus for understanding its function. It has been reported that Nur77 levels are regulated by both transcriptional and post-transcriptional mechanisms^11,12^. In addition, post-translational modifications have also been described as being an important mechanism for the regulation of Nur77 levels. Among post-translational modifications, phosphorylation is the most common mechanism that has been shown to regulate Nur77^13–16^. Interestingly, it is suggested that ubiquitination is also a possible mechanism for regulating Nur77 level^17^.

Protein degradation is one of the most important mechanisms for regulating protein levels, particularly for proteins with fast turnover. The reported half-life for Nur77 is extremely short, being of the order of 20–40 min^18^. Protein degradation mediated by ubiquitination is an important process that controls every aspect of cellular function. After sequential activation of ubiquitin by E1 and E2, E3 ligases transfer ubiquitin to internal lysine residues on target substrates to form mono ubiquitination or poly-ubiquitin. The specificity of ubiquitin transfer is conferred by ~ 600 E3 ligases in human cells. E3 ligases are classified based on the homologous to the E6AP carboxyl terminus (HECT), really interesting new gene (RING), or ring between ring fingers (RBR) domains. Studies on the interaction of Nur77 and ubiquitination have been previously reported by Hu *et al*. Celastrol promotes Nur77 ubiquitination at lysine 539 site by the mechanism of K63-linked chain formation by TNF receptor associated factor 2^19^. The polyubiquitination of Nur77 also reported the action of SUMO (small ubiquitin-like modifier)-dependent ring finger protein 4 (RNF4)^12^. Nevertheless, the precise mechanism of Nur77 ubiquitination still remains.

Before ubiquitination, some substrates for E3 ligases undergo phosphorylation to produce modified protein containing a “degron”. Similar to the case of E3 ligase, the identity of the kinase that regulates Nur77 ubiquitination is unclear. Given that both of these reversible covalent modifications have defined roles in altering the function of proteins it is inevitable that these two processes can have both positive and negative effects on each other^20^. For example, the Ser95-Pro motif phosphorylation by Pin1 has been shown to be critical for Nur77 regulation^13^. In this study, we chose to examine the phosphorylation of Nur77 by casein kinase 2 (CK2). Indeed, Nur77 contains PEST (proline, glutamic acid, serine, and threonine) sequences whose phosphorylation typically facilitates protein degradation^21^. Importantly, the putative PEST sequences in Nur77 also contain a consensus site for CK2 phosphorylation. CK2 is a pleiotrophic, highly conserved serine/threonine kinase that consists of two α catalytic and two β regulatory subunits^22^. CK2 is responsible for the phosphorylation of hundreds of substrates within the cell and controls many cellular processes^23^. It has been shown for example, that CK2 controls the stability of IκB (inhibitor of κB)^24^ and PML (promyelocytic leukemia) proteins^25^. In addition, van Tiel *et al*. reported the involvement of CK2 in Pin1-mediated Nur77 stability^26^.

The aim of the present study was to identify the specific E3 ligase required for Nur77 ubiquitination and to examine whether CK2 facilitates Nur77 degradation by promoting its phosphorylation. Herein, we report the identification of Trim13 as the E3 ligase that ubiquitinates Nur77. We show that Trim13 interacts with Nur77 and directly promotes its ubiquitination at lysine 539 (K539) and further degradation. We also show that phosphorylation of Nur77 at serine 154 (S154) by CK2α triggers Nur77 ubiquitination by Trim13 and subsequent degradation, resulting in the control IL-6 production in response to TNFα signaling.

## Results

### Nur77 is degraded by the ubiquitin-proteasome pathway

**Figure 1:**
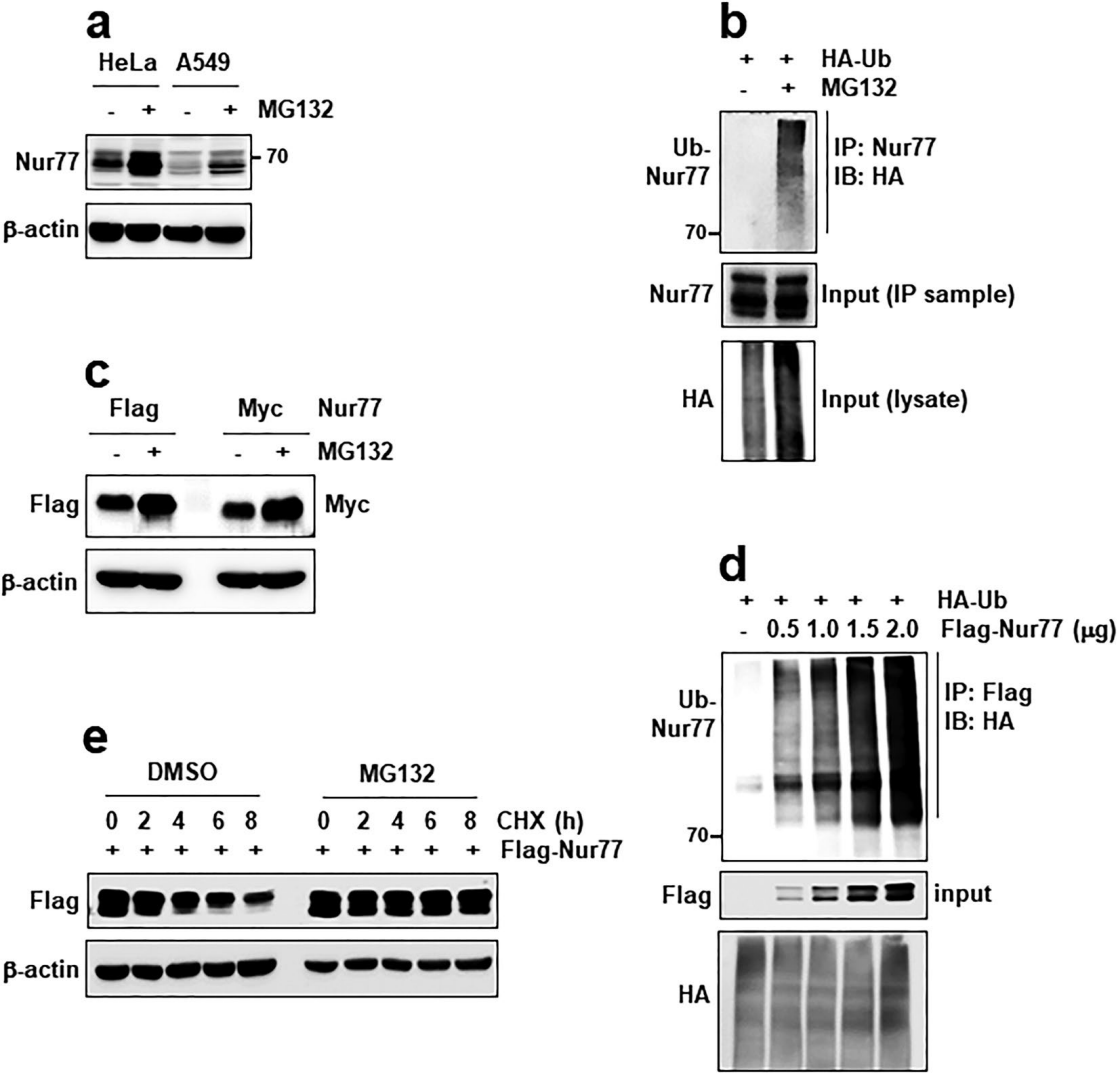
Nur77 undergoes degradation *via* the ubiquitin-proteasome pathway. (**a**) Cells were cultured in the absence or presence of MG132 (10 μM) for 6 h and Nur77 protein was detected by immunoblotting (IB). (**b**) HeLa cells were transfected with HA-ubiquitin (Ub) in the absence or presence of MG132 and analyzed by immunoprecipitation (IP) and IB as indicated. Five percent of the IP sample was analyzed by IB (lower panel). Ub-Nur77 = polyubiquitin chains of Nur77. (**c**) HeLa cells were transfected with Flag-tagged Nur77 or Myc-tagged Nur77 for 30 h. The cells were treated with MG132 as indicated and were analyzed by IB. (**d**) 293FT cells were transfected with the indicated amounts of Flag-Nur77 plasmids. The cell lysates were analyzed by IP and IB as indicated. Five percent of the cell lysates was analyzed by IB (lower panel). (**e**) 293FT cells were transiently transfected with Flag-Nur77 plasmid and treated with CHX (50 μg/ml) for the indicated times, in the absence or presence of MG132, and were analyzed by IB.

Nur77 is an orphan nuclear receptor that contains a predicted PEST sequence located at amino acid residues 32–104. PEST sequences are associated with proteins that are rapidly degraded^27^. We first examined the expression of Nur77 in nine cell lines using commercial antibodies. Among them, HeLa and A549 cell lines were found to highly express Nur77 compared to the other cell lines examined. However, the predicted Nur77 protein was represented by several bands, therefore we confirmed that these bands are Nur77 proteins by siRNA method (data not shown). We next determined whether Nur77 is degraded in two cell lines. Treatment of HeLa and A549 cell lines with the proteasome inhibitor MG132 led to an increased level of Nur77 (Fig. 1a). Based on these data, we hypothesized that Nur77 undergoes ubiquitin-mediated degradation in these cells. To test whether Nur77 does indeed undergo ubiquitination, we performed ubiquitination assay in HeLa cells. Immunoprecipitation (IP) of Nur77 followed by western blot analysis detected a ladder of HA-tagged Nur77 in the presence of MG132 (Fig. 1b). Because Nur77 ubiquitination signal was very weak, we confirmed Nur77 stability and ubiquitination in the overexpressed HeLa cells. Treatment with MG132 also increased Nur77 levels in the Flag-Nur77 or Myc-Nur77 overexpressed cells (Fig. 1c). We observed that Nur77 ubiquitination also clearly increased in a dose-dependent manner (Fig. 1d). Moreover, we examined Nur77 stability by cycloheximide (CHX) chase experiment. Treatment with CHX reduced levels of the Nur77 protein in a time-dependent manner. However, pretreatment with MG132 blocked this degradation of Nur77 (Fig. 1e). Taken together, we conclude that regulation of Nur77 turnover involves the ubiquitin-proteasome pathway.

### Trim13 is the E3 ligase required for Nur77 ubiquitination

**Figure 2:**
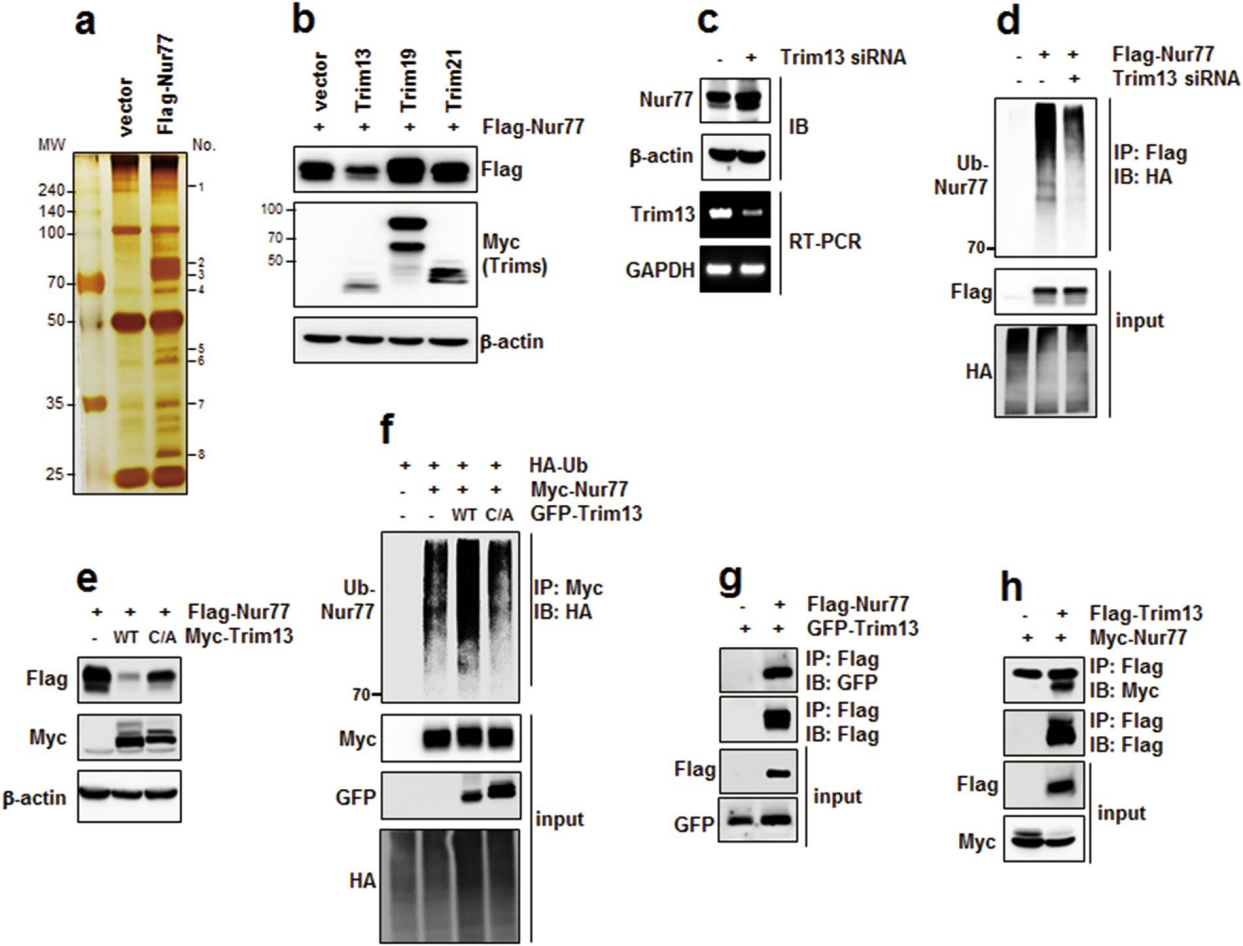
Trim13 is an E3 ligase for Nur77 ubiquitination. (**a**) 293FT cells were transfected with vector or Flag-Nur77 for 36 h, and the lysates were separated by SDS-PAGE followed by silver staining. Representative silver-stained images of the vector or Nur77 transfected cell lysates. (**b**) HeLa cells were transfected Myc-tagged Trim13, Trim19, or Trim21 along with the Flag-Nur77 plasmid and analyzed by IB as indicated. (**c,d**) HeLa cells were transfected with either control siRNA or Trim13 siRNA to access stability and ubiquitination. Nur77 protein was detected by IB and Trim13 mRNA was detected by RT-PCR. For ubiquitination of Nur77, the cell lysates were analyzed by IP and IB as indicated. (**e**) HeLa cells were co-transfected with Flag-Nur77 either WT Trim13, or its inactive mutant (C/A = C10/13A), and analyzed by IB. (**f**) HeLa cells were co-transfected with Nur77 and either WT Trim13 or its inactive mutant, to access ubiquitination and analyzed by IP and IB. (**g**) HeLa cells were transfected with Trim13 in the absence or presence of Flag-Nur77. The cell lysates were immunoprecipitated as shown, and the samples were subjected to electrophoresis, and analyzed by IB. (**h**) HeLa cells were transfected with Nur77 in the absence or presence of Flag-Trim13. The cell lysates were immunoprecipitated as shown, and the samples were subjected to electrophoresis, and analyzed by IB.

To identify the specific E3 ligase for Nur77 ubiquitination, we performed a comparative proteomics study in 293FT cells. The data in Fig. 2a show representative silver-stained images using vector or Flag-Nur77 overexpressed samples. By comparing these two lanes, ~10 bands were found to have different levels of expression, eight of which were successfully identified by LC-MS/MS analyses. The characteristics of the identified bands, including protein name, gene name, NCBI accession number, molecular mass, and protein score are summarized in Table 1. Among these identified proteins, two were related to ubiquitin E3 ligases; one was Trim13 and the other was Trim21. We cloned Trim13 or Trim21 into a Myc expression plasmid and assessed Nur77 stability. Overexpression of Trim13 strongly decreased Nur77 stability, whereas Trim21 did not affect Nur77 stability. We also tested the effect of Trim19, but it rather increased Nur77 stability (Fig. 2b). On the other hand, Trim13 knock-down by siRNA increased Nur77 stability and decreased their ubiquitination (Fig. 2c,d). The activity of Trim13 depends on the Cys10 and Cys13 (C10/13) residues which are active sites. Therefore, we constructed a double point mutant whereby the C10/13 residues were converted to A10/13 by site-directed mutagenesis and compared the stability of this Nur77 mutant with that of WT Trim13. Overexpression of WT Trim13 decreased Nur77 level, whereas C10/13A mutant partially recovered Nur77 stability (Fig. 2e). In addition, we observed that WT Trim13 increases Nur77 ubiquitination, on the other hand the C10/13A mutant was not (Fig. 2f). These results suggest that Trim13 activity mediates Nur77 ubiquitination, leading to its degradation. Next, we observed the interaction between Nur77 and Trim13 by immunoprecipitation. (Fig. 2g,h). Taken together, these data suggest that Trim13 is an important E3 ligase for Nur77 ubiquitination.

**Table 1:** List of the characteristics of the identified bands.

Identified band No.	Protein name	Gene name	NCBI accessionnumber	Molecular weight (Da)	Score
1	CAD protein	CAD	NM_001306079.1	245317	36
	Talin-1	TLN1	NP_006280.3	272024	17
2	Nuclear receptor subfamily 4 group A member 1	NR4A1	NM_001202233.1	65596	222
	Nuclear receptor subfamily 4 group A member 2	NR4A2	NP_006177.1	67636	117
	Heat shock protein HSP 90-beta	HSP90AB1	NP_001258898.1	83602	84
	Oxidative stress-induced growth inhibitor 2	OSGIN2	NP_001119583.1	57180	25
	Mitochondrial inner membrane protein	IMMT	NP_001093639.1	84094	23
	Coiled-coil domain-containing protein 87	CCDC87	NP_060689.2	96805	21
3	Nuclear receptor subfamily 4 group A member 1	NR4A1	NM_001202233.1	65596	174
	ATP-dependent RNA helicase DDX3X	DDX3X	NM_001193416.2	73640	66
	ATP-binding cassette sub-family F member 2	ABCF2	NM_005692.4	71862	32
	Eukaryotic translation initiation factor 4B	EIF4B	NM_001330654.1	69205	32
	Melanoma-associated antigen D2	MAGED2	NM_014599.5	65125	27
4	Heat shock 70 kDa protein 1A/1B	HSPA1A	AQY76873.1	70350	283
	Probable ATP-dependent RNA helicase DDX5	DDX5	NM_001320595.1	69680	37
	Protein arginine N-methyltransferase 5	PRMT5	NM_001039619.2	73378	29
	RNA-binding protein 39	RBM39	NM_001242599.1	59665	25
	Rho GTPase-activating protein 25	ARHGAP25	NM_001007231.2	73964	22
	Calcium-binding mitochondrial carrier protein Aralar2	SLC25A13	NM_001160210.1	74575	21
	Inositol-triphosphate 3-kinase C	ITPKC	NM_025194.2	75950	19
5	Elongation factor 1-alpha 1	EEF1A1	NM_001402.5	50477	49
	E3 ubiquitin-protein ligase TRIM13	TRIM13	NM_001007278.2	46988	30
	60 S ribosomal protein L4	RPL4	NM_000968.3	47984	22
	Monoacylglycerol lipase ABHD12	ABHD12	NM_001042472.2	45545	21
	E3 ubiquitin-protein ligase TRIM21	TRIM21	NM_003141.3	55207	18
6	Methylosome protein 50	WDR77	NM_001317062.1	36353	129
	Eukaryotic initiation factor 4A-I	EIF4A1	NP_001407.1	46393	93
	60S ribosomal protein L3 isoform a	RPL3	NP_000958.1	46392	57
	Cytochrome b-c1 complex subunit 2, mitochondrial	UQCRC2	NP_003357.2	48631	43
7	Zinc finger protein 778 isoform X3	ZNF778	XP_016878505.1	45940	27
	Basement membrane-specific heparan sulfate Proteoglycan core protein isoform b precursor	HSPG2	NP_005520.4	47979	22
8	ADP/ATP translocase 2	SLC25A5	NM_001152.4	33078	108
	ADP/ATP translocase 1	SLC25A4	NM_001151.3	33291	85
	BTB/POZ domain-containing protein KCTD5	KCTD5	NM_018992.3	26493	46
	Mitochondrial dicarboxylate carrier	SLC25A10	NM_001270888.1	31738	44
	Vesicle-associated membrane protein-associated protein B/C	VAPB	NM_001195677.1	27457	27
	Histone H1x	H1FX	NM_006026.3	22488	20

**Figure 3:**
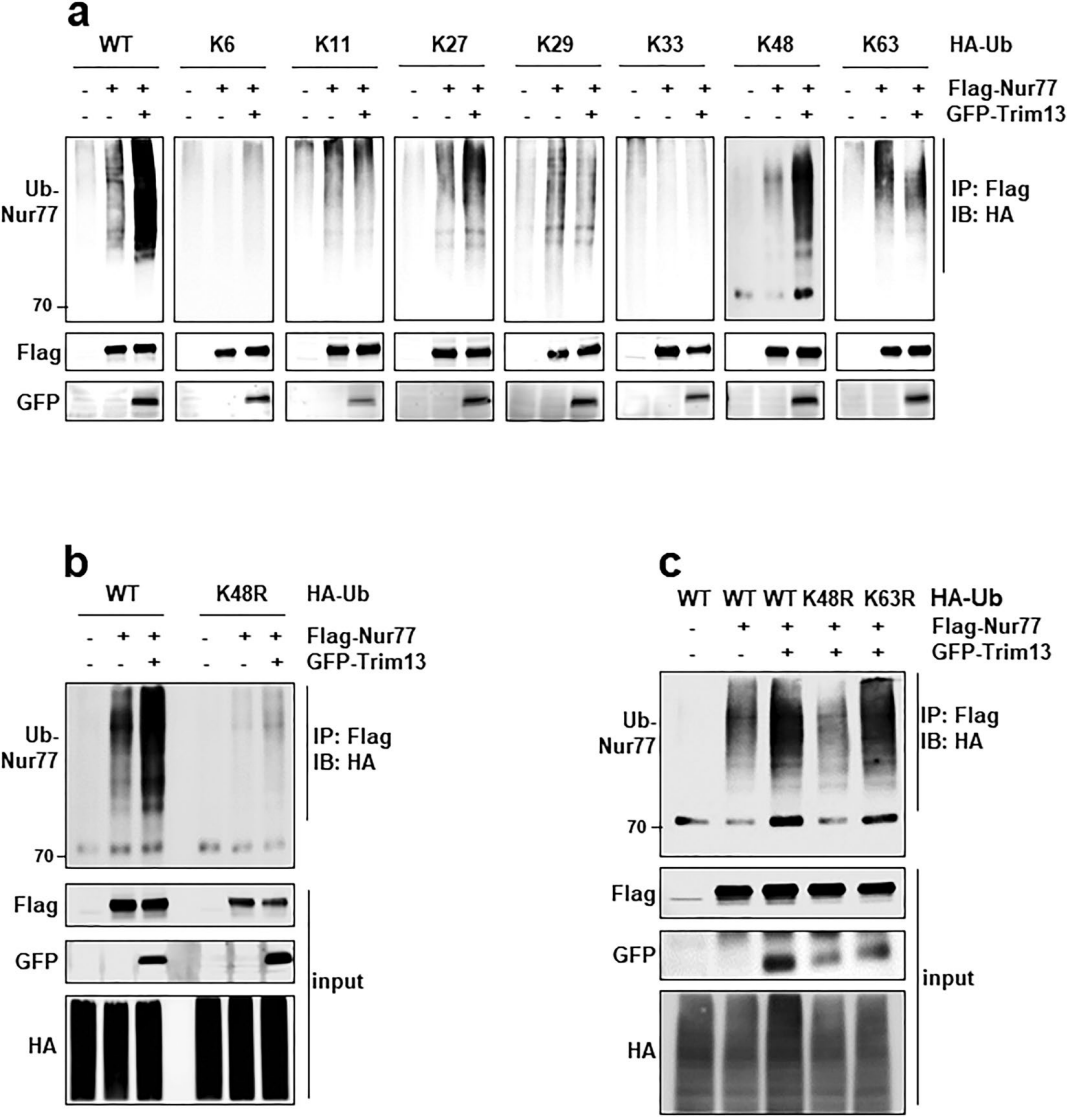
Trim13 ubiquitinates Nur77 *via* K48-linked chain formation. (**a**) HeLa cells were transfected with various types of HA-ubiquitin (WT or mutants) along with combination of Flag-Nur77 or GFP-Trim13. An ubiquitination assay was performed after 36 h, and the expression of samples was determined by IB. (**b**) HeLa cells were transfected with WT ubiquitin or the K48R mutant with a combination of Flag-Nur77 or GFP-Trim13 to access ubiquitination. The cell lysates were immunoprecipitated as shown, and the samples were subjected to electrophoresis, and analyzed by IB. (**c**) HeLa cells were transfected with WT ubiquitin, the K48R mutant, or the K63R mutant with a combination of Flag-Nur77 or GFP-Trim13 to access ubiquitination.

We analyzed the type of ubiquitin chain formed on Nur77 as a result of Trim13 activity. For this, we used a set of ubiquitin mutants in which all except one Lys residue was replaced by Arg. The ubiquitination of Nur77 itself was expected to have various chain formation including K27 and K63. The ubiquitination of Nur77 by Trim13 was most prominent in K48-linked chain formation (Fig. 3a). We used another ubiquitin mutants in which one Lys residue was replaced by Arg. Further experiment showed that the ubiquitination of Nur77 by Trim13 is dependent on K48-linked chain formation (Fig. 3b). However, the Nur77 ubiquitination by Trim13 was not associated with K63-linked chain formation (Fig. 3a,c).

### Lys539 site is responsible for ubiquitination of Nur77 by Trim13

**Figure 4:**
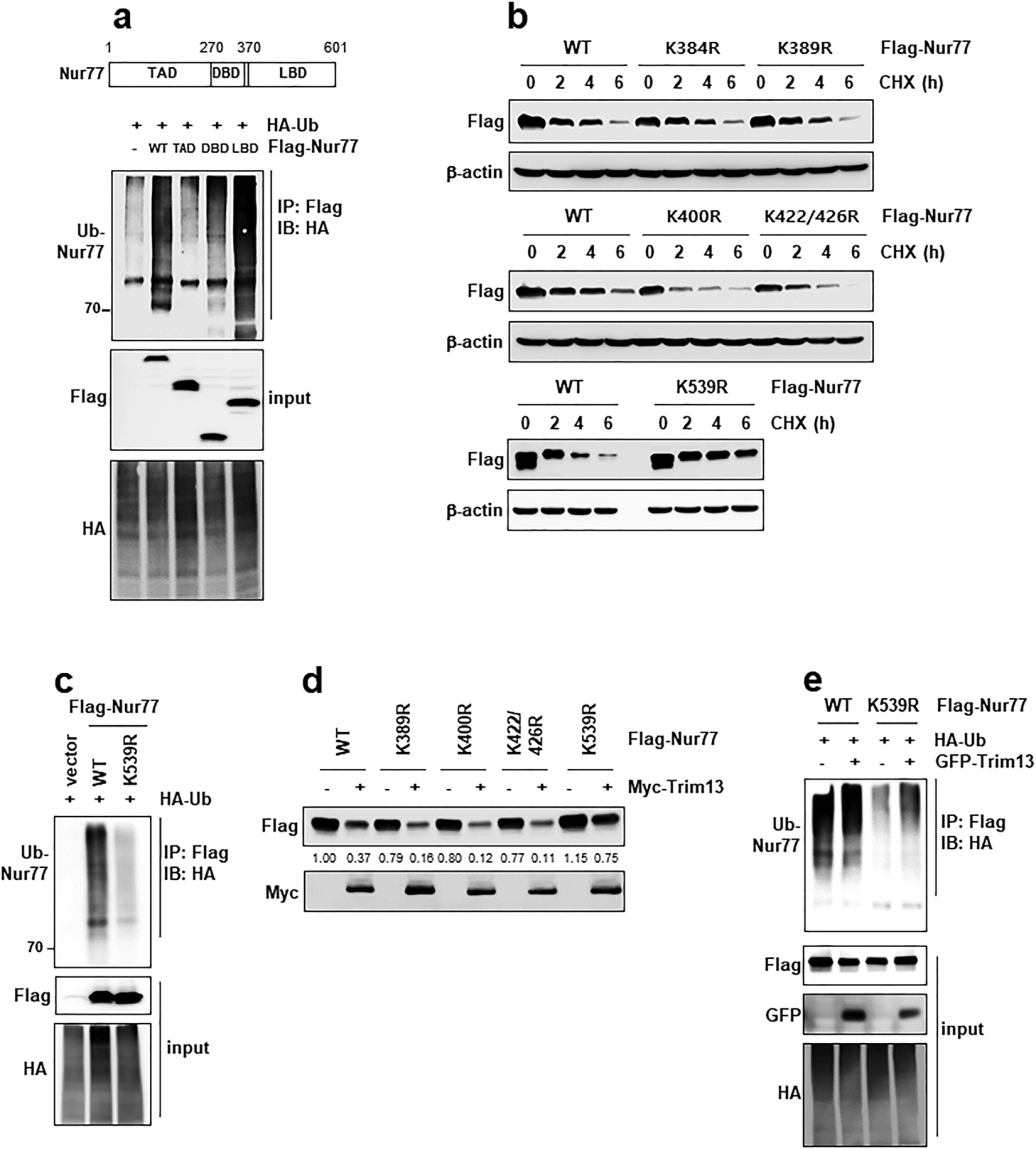
K539 residue is important for Trim13-mediated Nur77 ubiquitination and degradation. (**a**) 293FT cells were transfected with either WT Nur77, or its domain mutants as indicated, and the lysates were analyzed by IP and IB as indicated. Ten percent of the input lysate was analyzed by IB with a Flag antibody. Schematic representation of Nur77 constructs used in this study (upper scheme). WT, wild type; TAD, transactivation domain; DBD, DNA-binding domain; LBD, ligand-binding domain. (**b**) Cells were transfected with WT Nur77 or its mutants and then treated with CHX for the indicated times, following which lysates were analyzed by IB. (**c**) Cells were transfected with either WT Nur77 or K539R to access ubiquitination and analyzed by IP and IB as indicated. (**d**) Cells were co-transfected with WT Nur77, or indicated mutants, in the absence or presence of Trim13. The protein levels of Nur77 and Trim13 were evaluated. Nur77 levels were quantified by densitometry and the values determined are shown under each corresponding band. (**e**) HeLa cells were co-transfected with WT Nur77, or its K539R mutant, in the absence or presence of Trim13 to access ubiquitination and analyzed by IP and IB as indicated.

To find the specific site in the Nur77 ubiquitination, we generated a series of domain-containing mutants. As a references, the structure of Nur77 is presented in Fig. 4a (upper panel). Transfection with the putative ligand-binding domain (LBD) resulted in an ubiquitination of Nur77, whereas transactivation domain (TAD) did not change the ladder formation. DNA-binding domain (DBD) resulted in a slight increase ubiquitination that was clearly less than that seen with WT Nur77 (Fig. 4a). These data suggest that the site of Nur77 ubiquitination lies in the LBD. To identify precisely the site ubiquitinated by Trim13, we analyzed the protein sequence of Nur77 and found several lysine sites predicted to be likely ubiquitination sites. Most of the putative sites are located in the putative LBD, but some are located in the TAD. Based on the above, we generated five Nur77 mutants with an arginine (R) in place of each lysine (K) to identify target site. We then performed a CHX chase experiment to examine the effects of the mutants. Mutation of four sites (K422/426 R is double mutant) did not change Nur77 stability, whereas K539R did affect its stability (Fig. 4b). In addition, K539R mutant decreased the ubiquitination of Nur77 (Fig. 4c). Based on these data the K539 residue appears to be a specific site for Nur77 ubiquitination. This Lys site was consistent with the results of Hu *et al*.^19^. Next, we tested the role of K539 in the Trim13-mediated Nur77 stability. The K539R mutant significantly blocked degradation and ubiquitination of Nur77 by Trim13 (Fig. 4d,e). These data suggest that the K539 is important site for Trim13-mediated Nur77 ubiquitination.

**Figure 5:**
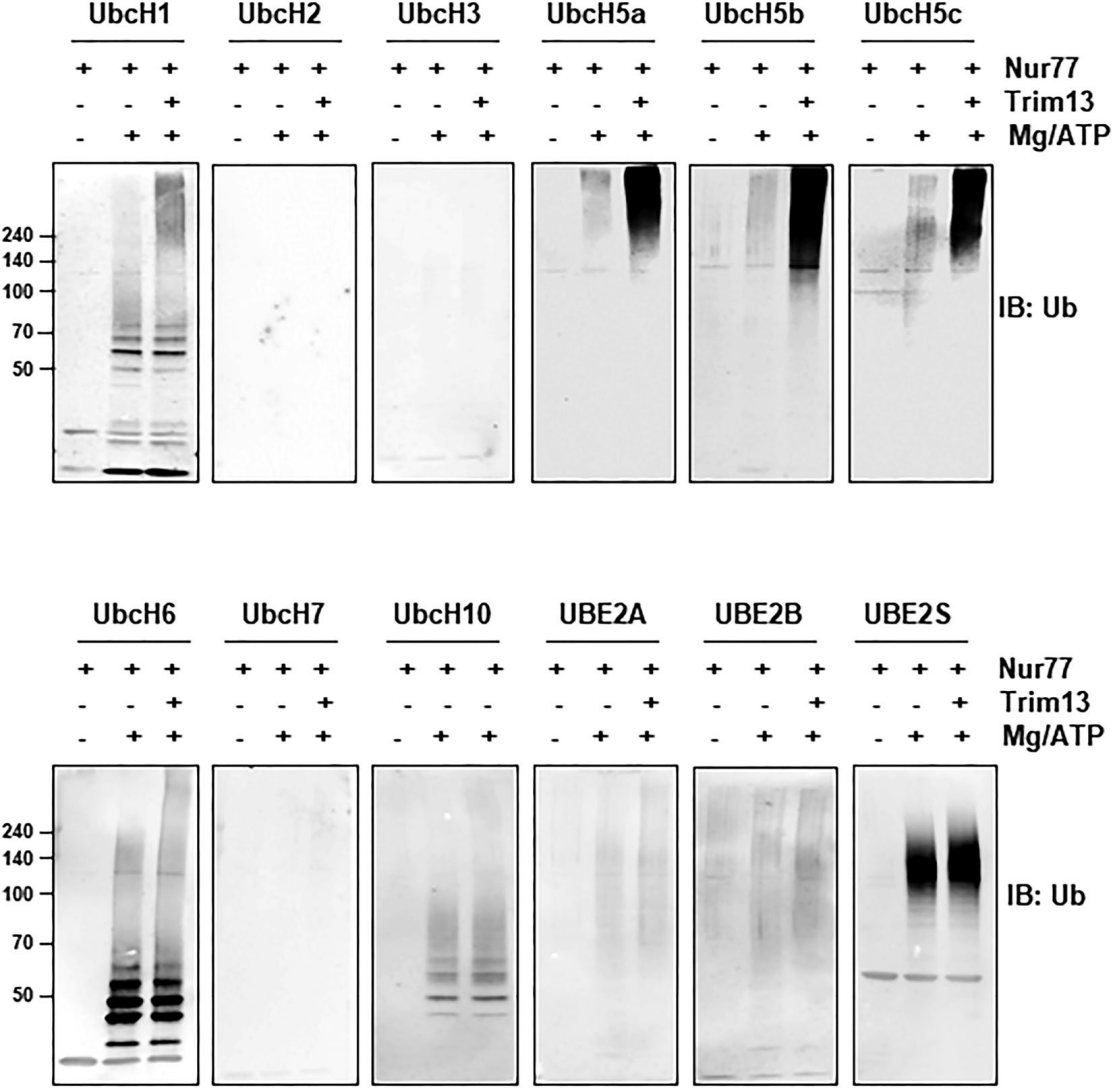
E2 UbcH5 is involved in Trim13-mediated Nur77 ubiquitination. 293FT cells were transfected with vector or Flag-Trim13, after which the Trim13 protein was purified using Flag affinity beads. An *in vitro* ubiquitination assay was then performed with various combinations of a mixture of E1 enzyme, different E2 enzymes, ATP, ubiquitin, recombinant Nur77 and Flag-Trim13 proteins.

An *in vitro* ubiquitination assay was performed to determine which E2 was involved in the Nur77 ubiquitination by Trim13. First, we obtained recombinant Trim13 protein from 293FT cells. A mixture containing recombinant Nur77, Flag-Trim13, E1 and different E2 enzymes was then incubated with ubiquitin in the absence or presence of Mg/ATP. Ubiquitination of Nur77 itself was increased by the UBE2S. However, Nur77 ubiquitination by Trim13 was optimal with UbcH5 among the E2 species tested; there was no difference between UbcH5 isoforms (Fig. 5). These data suggest that the E2 UbcH5 is involved in Trim13-mediated Nur77 ubiquitination.

### CK2α is responsible for degradation of Nur77

**Figure 6:**
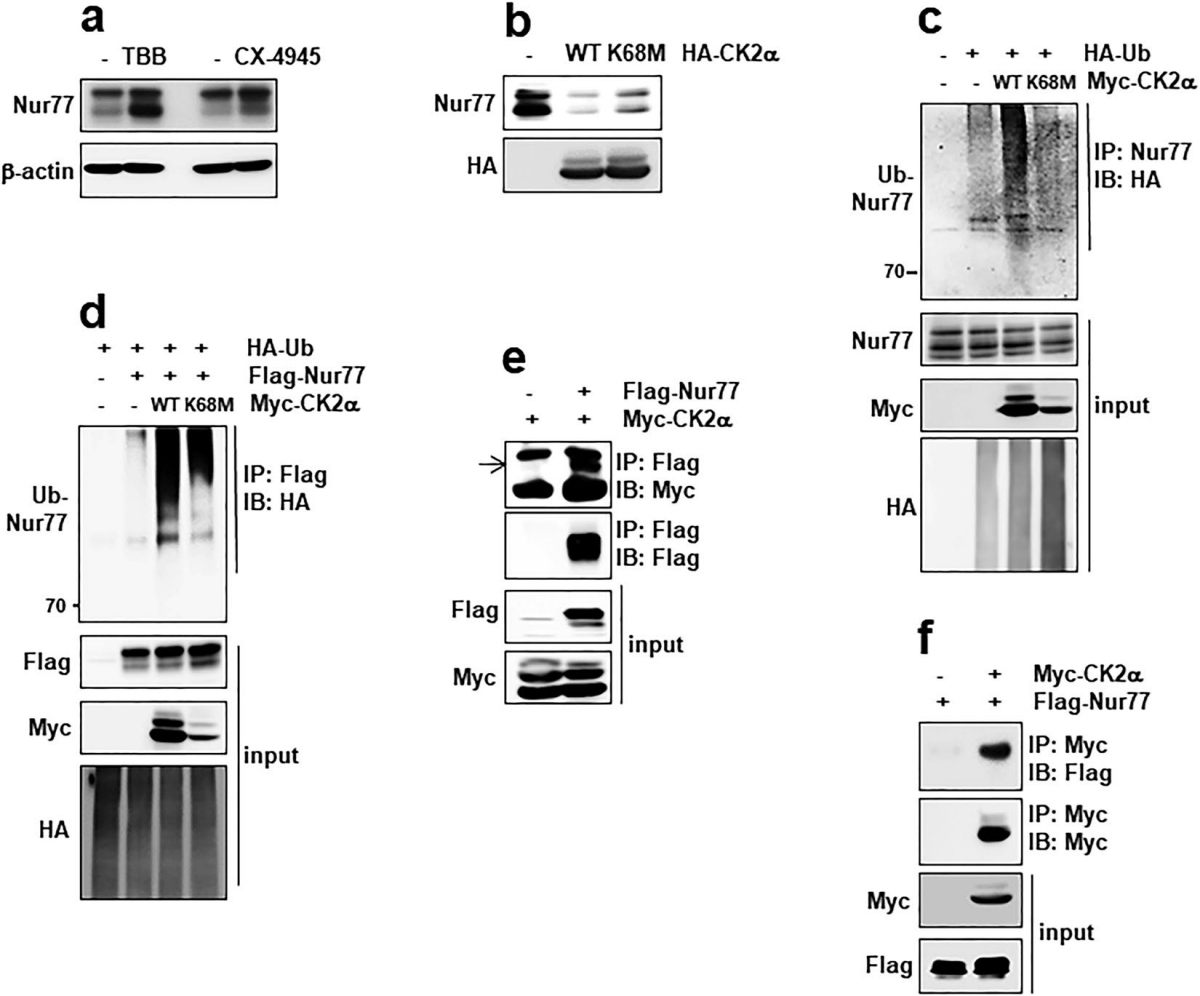
CK2α interacts with Nur77 and regulates its ubiquitination. (**a**) HeLa ells were treated with TBB (50 μM) or CX-4945 (10 μM) for 6 h and the levels of Nur77 were measured. (**b**) HeLa cells were co-transfected with WT Myc-CK2α, or its inactive mutant (K68M), and Nur77 and analyzed by IB. (**c**) HeLa cells were transfected with WT CK2α, or its inactive mutant (K68M), to access Nur77 ubiquitination and were analyzed by IP and IB as indicated. (**d**) Nur77 was co-transfected along with either WT CK2α, or its inactive mutant (K68M) to access ubiquitination and were then analyzed by IP and IB as indicated. (**e**) HeLa cells were transfected with Myc-CK2α in the absence or presence of Flag-Nur77. The cell lysates were immunoprecipitated with the indicated antibodies and the samples were subjected to electrophoresis, and analyzed by IB. (**f**) HeLa cells were transfected Flag-Nur77 in the absence or presence of Myc-CK2α. The cell lysates were immunoprecipitated with the indicated antibodies and the samples were subjected to electrophoresis, and analyzed by IB.

Nur77 is a phosphoprotein that contains various putative phosphorylation sites for kinases such as c-jun N-terminal kinase, mitogen-activated protein kinase 1 (MAPK1), CK2, protein kinase A, and Akt^15,21,26,28,29^. To address the potential role of CK2 in Nur77 degradation, cells were treated with TBB (4,5,6,7-tetrabromobenzotriazole) or CX-4945 (Silmitasertib), specific inhibitors of CK2. Treatment with TBB or CX-4945 increased Nur77 protein levels (Fig. 6a). To confirm whether CK2 participates in Nur77 stability, WT CK2α and its mutant (K68M in the CK2α subunit, a kinase-dead form) were overexpressed in HeLa cells. Nur77 level was decreased in the cells overexpressing WT CK2α. However, its level was partially recovered in the cells overexpressing the K68M mutant (Fig. 6b). Furthermore, WT CK2α increased Nur77 ubiquitination, but had a lesser effect on the K68M mutant (Fig. 6c,d). Next, we tested the interaction between Nur77 and CK2α using immunoprecipitation with anti-Flag bead (Nur77) or anti-Myc antibody (CK2α). The data showed that CK2α interacts with Nur77 (Fig. 6e,f). These data suggest that CK2α interacts with Nur77 and regulates its ubiquitination.

### The Ser154 residue in Nur77 is a target site for CK2α

**Figure 7:**
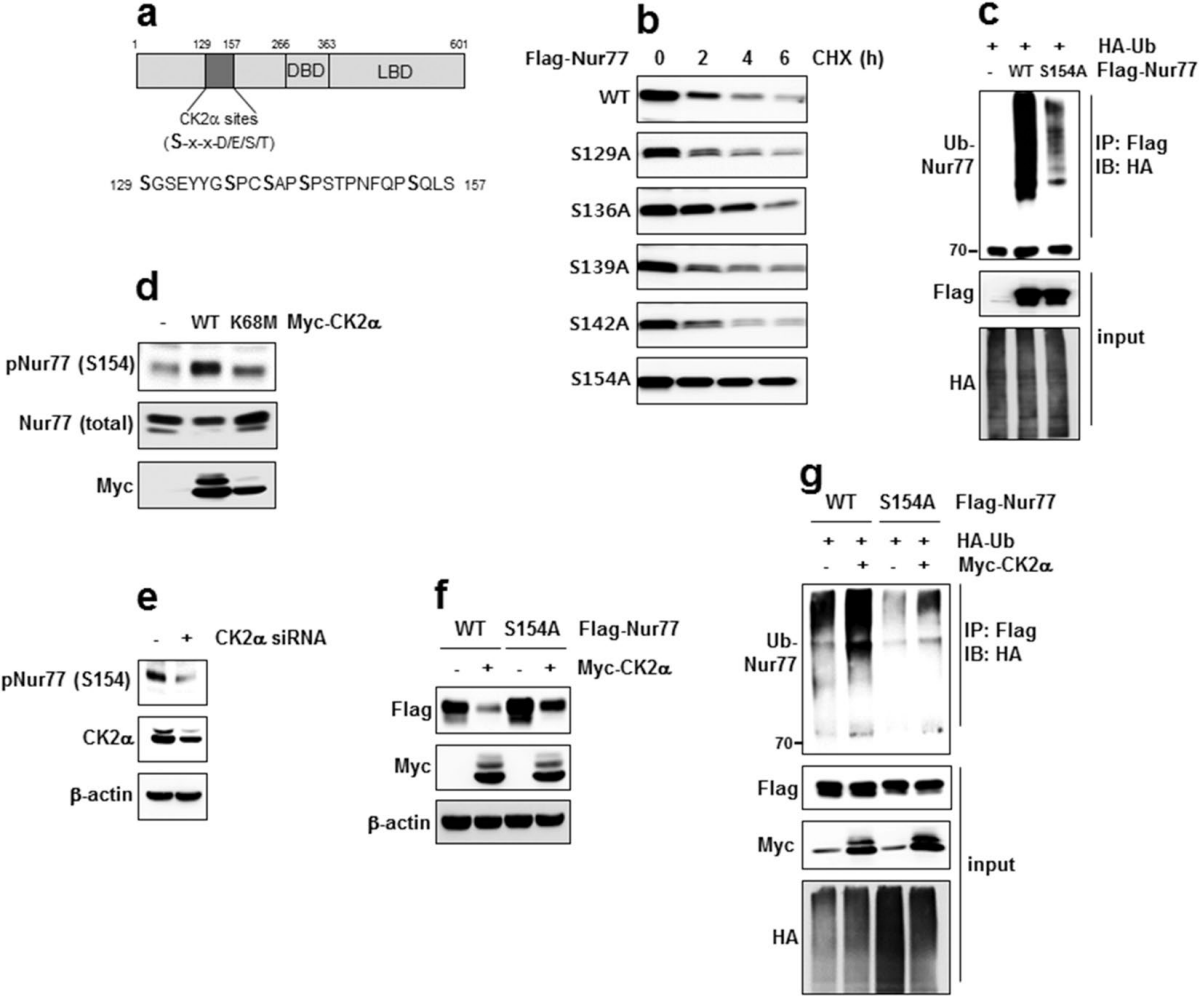
S154 of Nur77 is a target for CK2α-mediated phosphorylation and stability. (**a**) Schematic representation of the Nur77 protein. Bold characters, putative CK2α-phosphorylation sites. (**b**) WT Nur77 or various point mutants were transfected into cells and treated with CHX for the indicated times. The protein levels of Nur77 were evaluated by IB. (**c**) HeLa cells were transfected with either WT Nur77, or the S154A mutant to access ubiquitination and were then analyzed by IP and IB as indicated. Ten percent of the input lysate was analyzed by IB. (**d**) Nur77 was co-transfected along with either WT CK2α or K68M mutant to access phosphorylation, and were then analyzed by IB using phospho-Nur77 (pSer154) or Nur77 (total Nur77) antibody. (**e**) HeLa cells were transfected with either control siRNA or CK2α siRNA to access Nur77 phosphorylation and analyzed by IB as indicated. (**f**) Cells were transfected with either WT Nur77, or the S154A mutant, in the absence or presence of CK2α to access ubiquitination and were analyzed by IP and IB as indicated. (**g**) HeLa cells were transfected with either WT Nur77, or the S154A mutant, in the absence or presence of CK2α to access stability and were analyzed by IB as indicated.

A motif scan analysis revealed the presence of the putative CK2 target sites in the N-terminal of Nur77. Moreover, a computer alignment indicated that there were multiple sites within the 129–157 region of Nur77 that contained the potential optimal CK2 substrate motif (S-x-x-E/D/S/T) (Fig. 7a). To identify the exact CK2α target site, we generated point mutants at five serine residues (S129, S136, S139, S142 and S154) within the 129–157 region of Nur77 and performed a CHX chase assay. Of the five mutants, the S154A mutant most strongly affected the stability of Nur77. On the other hand, S129A, S139A and S142A mutants were not affected and the S136A mutant was less effective than the S154A mutant (Fig. 7b). To confirm that S154 residue is important for the ubiquitination of Nur77, the WT Nur77 or S154A mutant were transfected into cells. The mutant exhibited reduced ubiquitination compared with that of WT Nur77 (Fig. 7c). To prove that S154 residue of Nur77 is directly phosphorylated by CK2α, we made an antibody that is specific for Nur77 phosphorylated at S154. Although the S154 phosphorylation of Nur77 appeared in the vector-transfected cells, the transfection of CK2α increased these phosphorylation but the K68M mutant did not (Fig. 7d). CK2α knock-down by siRNA reduced the phosphorylation of S154 in Nur77 (Fig. 7e). These result implies that CK2α directly phosphorylates S154 of Nur77. Next, we confirmed the importance of S154 residue in CK2α-mediated Nur77 ubiquitination and stability. The expression of the S154A mutant partially blocked not only the ubiquitination but also the degradation of Nur77 (Fig. 7f,g). These results suggest that S154 phosphorylation of Nur77 by CK2α regulates Nur77 ubiquitination and degradation.

### TNFα stimulates Nur77 degradation via CK2α-Trim13 pathway

**Figure 8:**
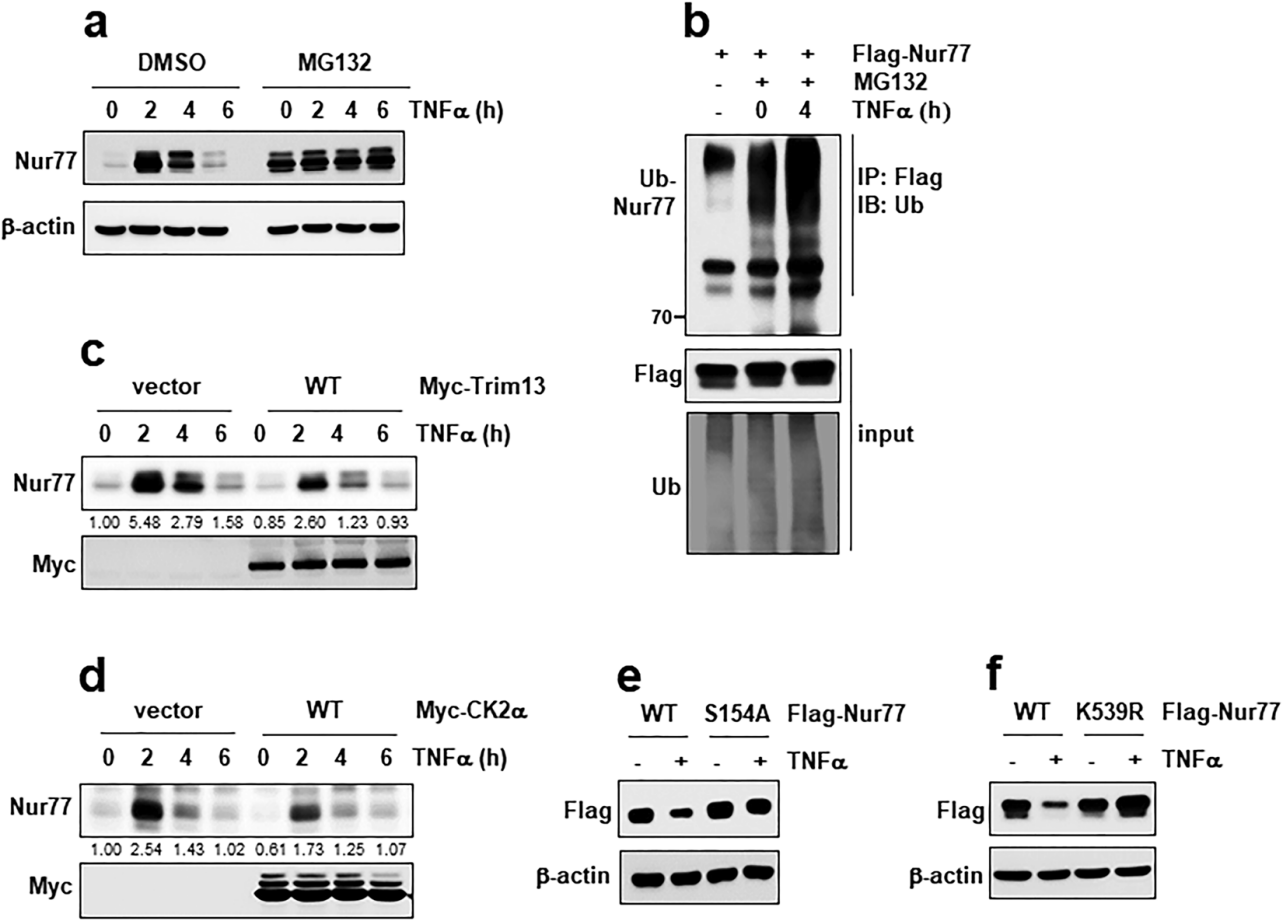
TNFα stimulates Nur77 degradation by ubiquitin-proteasome pathway. (**a**) HeLa cells were treated with TNFα (5 ng/mL) for the indicated times, in the absence or presence of MG132 (10 μM). The protein levels of Nur77 were evaluated by IB. (**b**) HeLa cells were transfected with Nur77, and then treated with vehicle or TNFα for 4 h to access ubiquitination and analyzed by IP and IB as indicated. (**c**) HeLa cells were transfected with vector or CK2α, and then treated with TNFα for the indicated times. (**d**) HeLa cells were transfected with vector or Trim13, and then treated with TNFα for the indicated times. The protein levels of Nur77 were evaluated by IB. (**e**) HeLa cells were transfected with either WT Nur77, or the S154A mutant, and treated with vehicle or TNFα. (**f**) HeLa cells were transfected with either WT Nur77, or the K539R mutant and treated with vehicle or TNFα. The protein levels of Nur77 were evaluated by IB.

To determine the physiological significance of Nur77 degradation, we attempted to identify specific signal that mediate this phenomena and found TNFα. TNFα activity is a typical inflammation inducer in many cells. Indeed, TNFα treatment dramatically induced Nur77 protein as well as mRNA in HeLa cells, but these protein was rapidly degraded afterwards. However, pretreatment with MG132 sustained TNFα-induced Nur77 degradation (Fig. 8a). In addition, TNFα treatment increased the ubiquitination of Nur77 (Fig. 8b). These results suggest that the ubiquitin-proteasome pathway exists in TNFα-induced Nur77 regulation. Next we tested that effects of Trim13 and CK2α on Nur77 degradation mediated by TNFα. Overexpression of CK2α accelerated TNFα-induced Nur77 degradation (Fig. 8c). Similarly, TNFα-induced Nur77 proteins were degraded faster in Trim13- transfected cells than in vector-transfected cells (Fig. 8d). Taken together, these results suggest that both CK2α and Trim13 were involved in degradation of Nur77 mediated by TNFα. To examine the importance of S154 (phosphorylation site) and K539 (ubiquitination site) residues in TNFα-mediated Nur77 degradation, we compared stability in response to TNFα. The degradation of Nur77 induced by TNFα was ablated in the S154A or K539R mutants (Fig. 8e,f) suggesting that both the S154 and K539 residues are important for TNFα-mediated Nur77 stability.

### Nur77 suppresses TNFα-induced IL-6 production

**Figure 9:**
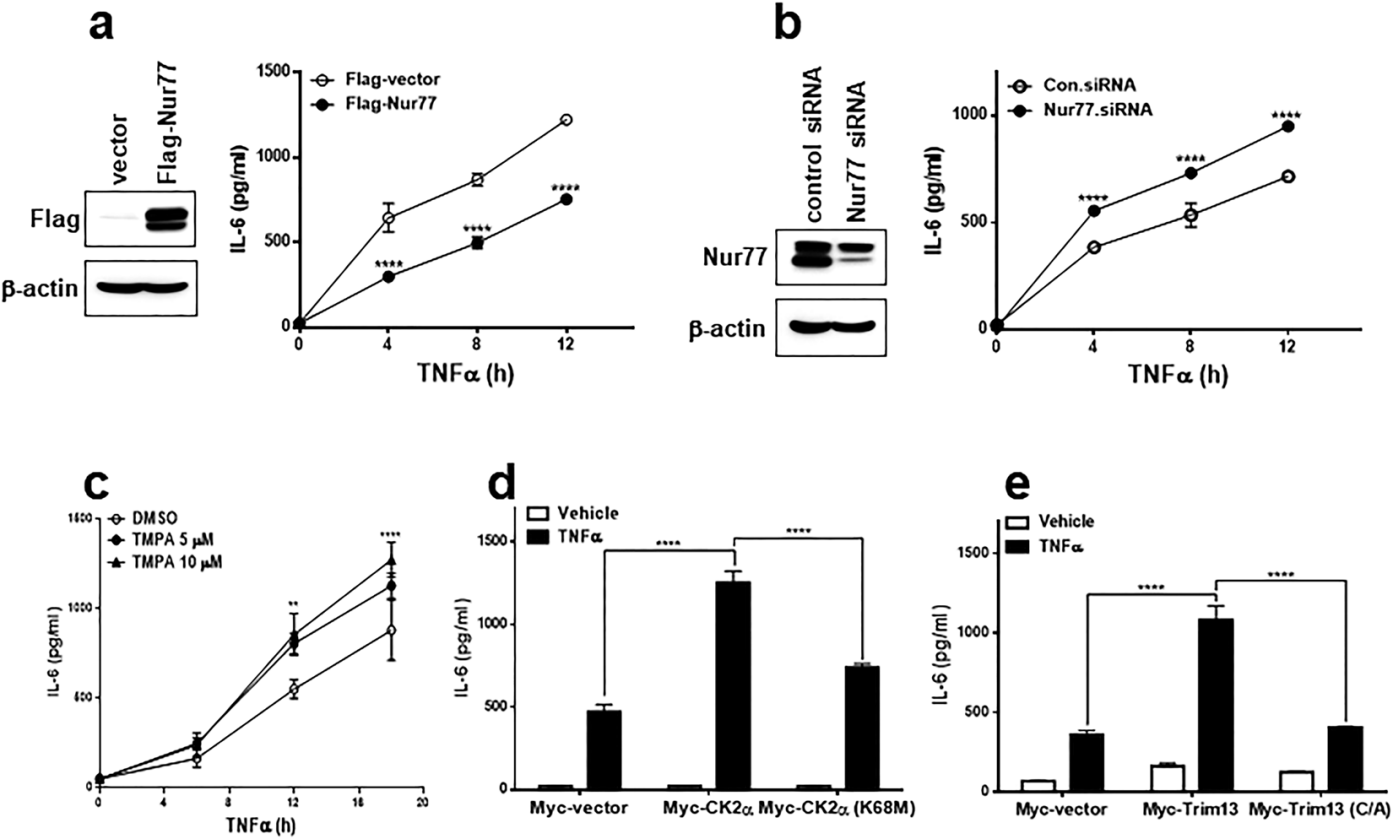
Nur77 inhibits TNFα-induced IL-6 production *via* CK2α-Trim13. (**a**) HeLa cells were transfected with either vector or Nur77 and treated with vehicle or TNFα (5 ng/mL). The cell lysates were analyzed for Nur77 by IB, and the supernatants were assayed for IL-6 using EIA. (**b**) HeLa cells were transfected with either control siRNA or Nur77 siRNA and treated with vehicle or TNFα. The cell lysates were analyzed for Nur77 by IB, and the supernatants were assayed for IL-6 using EIA. (**c**) HeLa cells were treated with TNFα for the indicated times in the absence or presence of different TMPA concentrations. The cell supernatants were assayed for IL-6 using EIA. (**d**) HeLa cells were transfected vector, CK2α, or the K68M mutant, and treated with vehicle or TNFα. The supernatants were assayed for IL-6 using EIA. (**e**) HeLa cells were transfected vector, Trim13, or its inactive mutant (C10/13A), and treated with vehicle or TNFα. The supernatants were assayed for IL-6 using EIA. **P < 0.01, ****P < 0.0001. The representative experiments were repeated in triplicate with similar results.

One important role of Nur77 is to control inflammation. Recently, Nur77 has been reported to exhibit an anti-inflammatory activity^19^. We therefore investigated the role of Nur77 in the TNFα-induced IL-6 production. Overexpression of Nur77 inhibited TNFα-induced IL-6 production, whereas knock-down of Nur77 using a targeted siRNA further increased TNFα-induced IL-6 production (Fig. 9a,b). Treatment of the Nur77 inhibitor, ethyl 2-[2,3,4-trimethoxy-6-(1-octanoyl)phenyl]acetate (TMPA) also further increased TNFα-induced IL-6 production (Fig. 9c). Next, we examined the relationship between Nur77 and IL-6 production based on the effect of CK2α or Trim13 on TNFα treatment. Transfection of CK2α potentiated TNFα-stimulated IL-6 production compared to vector, whereas transfection of the K68M mutant did not (Fig. 9d). Trim13 showed similar effects to CK2α. In other words, transfection of Trim13 potentiated TNFα-stimulated IL-6 production, whereas transfection of the C10/13A mutant did not (Fig. 9e). Taken together, those data suggest that changes in Nur77 levels, effected by CK2α or Trim13, play a role in the production of IL-6 in response to TNFα.

## Discussion

In the present study, using proteomic approach, we have shown that Nurr77 is ubiquitinated by the E3 ligase Trim13 leading to its degradation, and furthermore have uncovered the regulatory pathway that controls this ubiquitination. The K539 residue in Nur77 was shown to be the site of ubiquitination mediated by the E3 ligase Trim13. In parallel, the protein kinase CK2α was shown to be able to phosphorylate Nur77. We demonstrated that phosphorylation of S154 by CK2α in Nur77 is important for its stability. Furthermore, physiologically, Nur77 is degraded by ubiquitination in response to TNFα stimulation. The possibility of regulating Nur77 stability through ubiquitination has been previously suggested^17^, but not many studies has successfully identified the actual E3 ligase involved. Nur77 contains a putative PEST sequence located at its N-terminus, which is a potential proteolytic cleavage site. Our initial goal was to identify the E3 ligase that could stimulate Nur77 ubiquitination. Using a proteomics analysis both Trim13 and Trim21 were identified as potential E3 ligases capable of binding Nur77, Trim13 was able to modulate Nur77 stability in cells but Trim21 was not. As part of this study, we observed that Trim28, another Nur77 binding protein, as well as Trim19 and Trim21 was not able to decrease Nur77 stability (data not shown). Very recently the protein inhibitor of activated STAT3 (PIAS3) has been reported as being a SUMO E3 ligase that can promote SUMOylation and degrade Nur77^12^. On the other hand, Smad ubiquitination regulatory factor 1 (Smurf1) has been reported to be able to inhibit Nur77 degradation^30^. Therefore, these reported data suggest that various kinds of E3 ligases are involved in Nur77 ubiquitination through different mechanisms.

Trim13, a member of the Trim family, has been reported to play a role in diverse cellular functions such as cell death^31^, cancer^32^, and immunity^33^. Trim13 was firstly identified as a RING E3 ligase and plays a role in endoplasmic reticulum (ER)-associated degradation^34^. Further studies have shown that Trim13 regulates autophagy caused by ER stress^35^. To date, L-type channels, Akt, and caspase-8 have been shown to be substrates for Trim13-mediated ubiquitination^31,35,36^. Nevertheless, the specific substrates for Trim13 are still not very well characterized. Trim13, along with Trim59, is a unique E3 ligase since it contains a transmembrane domain, and is known to target only a few substrates. The active sites of Trim13 contains two cysteine residues (Cys10 and Cys13) that are important for ubiquitination of substrate. In this study, we have conclusively demonstrated that the ubiquitination and degradation of Nur77 are dependent on this Trim13 ligase activity. Using domain deletion mutants, we were able to identify the Nur77 ubiquitination site as being in its putative LBD, and through further detailed point mutation analyses, we found K539 was the target lysine ubiquitinated by Trim13. As we mentioned results, this Lys site was consistent with the results of Hu *et al*.^19^. It should be noted, however, that the K539R mutant was not able to completely inhibit Nur77 ubiquitination, suggesting that more target sites exist within Nur77.

Nur77, along with Nurr1 and Nor1, is an orphan nuclear receptor of the Nr4A family which has been recently studied as therapeutic target for inflammation^37^. The Nur77 receptor is rapidly induced by various inflammatory stimuli, suggesting that it has an important role in the inflammatory response. However, the mechanism by which Nur77 functions is not clear, principally because its ligands remain to be discovered. The Nur77 receptor has a bulky hydrophobic residue in place of the ligand-binding pocket that is usually found in other nuclear receptor LBDs^38,39^. Thus, it is also believed Nur77 does not bind a ligand, but rather functions as a ligand-independent transcription factor. There have however been several reports where synthetic molecules can act both as agonists or antagonists through direct binding to the LBD^40^, therefore it is still possible that a ligand exists. Regardless of the issue concerning its ligand, it appears that transcriptional regulation is the most well described function of Nur77^41^. Post-translational modifications, such as phosphorylation have also been suggested to be important regulators of Nur77 function^42^. Our study has convincingly shown that ubiquitination of Nur77 by an E3 ligase is also an important mechanism in regulating its activity. In addition, a very recent study has shown that SUMOylation may also be important in regulating Nur77^12^. Therefore, it is important to identify these various regulatory mechanisms that control Nur77 stability in order to fully understand its physiological function.

The most important finding of this study is that the connection between phosphorylation and ubiquitination acts as an essential mechanism regulating Nur77 stability. Our results suggest that phosphorylation on a specific Nur77 serine residue promotes Nur77 ubiquitination, further decreasing its stability. The possibility of Nur77 being regulated by phosphorylation has been suggested previously, and many putative target sites capable of being phosphorylated by kinases have been described, including phosphorylation of S97 by JNK, T145/S434 by MAPK1, S166 by DNA-PK, S344 by PKA, S354 by either S6K or Akt, etc.^15,21,26,28,29^. However, the connection between a specific Nur77 kinase and a specific Nur77 E3 ligase has not been made. We mutagenized many potential phosphorylation sites and examined their effect on Nur77 stability. Uniquely, mutagenesis of S154 was able to affect Nur77 stability. Based on this, we believe phosphorylation of S154 is a crucial for regulating Nur77 stability. It should be pointed out that mutation of T145, which is known to be phosphorylated by MAPK1, also had a slight effect on Nur77 stability; therefore, we are currently studying the role of MAPK1 in regulating Nur77 stability.

A motif scan analysis indicated that T55, S129, and S154 are potential sites for CK2. However, there were five putative target serine residues that are located between residues 129 and 157. When each individual serine was mutated to alanine, the S154 mutation had the strongest effect on Nur77 stability. These data therefore suggest that S154 phosphorylation by CK2α is important in controlling Nur77 stability. The CK2α phosphorylation site that we have identified in Nur77 is highly conserved across species including human, mouse, rat, bovine, and dog. However, alignment of this site across the two other Nur77 isoforms, Nurr1 and Nor1, did not reveal a consensus sequence. This suggest that the ubiquitination mechanism differs depending on the Nr4A isoform. Currently, it is estimated that CK2 has over 300 substrates that it can phosphorylate, and included among those are IκB, p21, p53, β-catenin, and breast cancer type 2 susceptibility protein 1 (BRCA1), which are all known to be regulated by a degradation mechanism^23^. Therefore, we believe Nur77 is also part of this group of CK2 substrate proteins that are regulated.

One of the most important functions of Nur77 is in the regulation of inflammation. Nur77 was initially identified as a pro-inflammatory factor, which was found to be abnormally overexpressed in inflamed synovial tissues, atherosclerosis, and multiple sclerosis^10,43^. In addition, pro-inflammatory stimuli such as lipopolysaccharide were known to also induce the expression of Nur77^44^. On the other hand, Nur77 acted as an anti-inflammatory in macrophages, epithelial cells, and endothelial cells, and therefore has been suggested to have anti-inflammatory actions^45–47^. A lack of Nur77 has been shown to increase inflammation, and there have been reports showing that Nur77 protects against lipopolysaccharide-induced sepsis^9^, also supporting the idea that Nur77 is an anti-inflammatory regulator. Our data shows that Nur77 overexpression inhibited TNFα-induced IL-6 production, whereas Nur77 knock-down increased IL-6 production. These results support that Nur77 could indeed act as an anti-inflammatory regulator. We believe that although TNFα increases Nur77 expression, and after a certain time Nur77 becomes degraded, decreasing the levels of this anti-inflammatory molecule, eventually causing elevated inflammation. Therefore, it is possible that the Nur77 level is important as an indicator of inflammation in cells. However, there is insufficient information on why TNFα drastically increases Nur77 expression. One hypothesis is that the expression of Nur77 is increased as part of defensive mechanism during the acute inflammatory response, but further mechanistic studies will be needed to fully understand this process.

## Materials and Methods

### Plasmids

Full-length mouse Nur77 cDNA was cloned into the pFLAG-CMV2 vector. For Myc-tagged Nur77, *Nur77* was amplified from pCMV2 Flag-tagged Nur77 by PCR and inserted into the EcoRI/BamHI sites. Full-length mouse Trim13 cDNA was also cloned into pFLAG-CMV2, pEGFP-C1, or pCMV2-Myc vectors. In order to construct Myc-tagged CK2α, *CK2*α was amplified from pRC/CMV-HA-CK2α by PCR and inserted into the EcoRI/BglII sites. Wild-type pRC/CMV-HA-CK2α and its mutant (K68M) were purchased from Addgene (Cambridge, MA). For Myc-tagged CK2α, *CK2*α was amplified from pRC/CMV HA-tagged CK2α by PCR and inserted into the EcoRI/BglII sites. Site-directed mutagenesis (C → A, S → A, or K → R) was performed using the Quick change mutagenesis kit and verified by performing DNA sequencing (Agilent branch, Daegu, South Korea). Recombinant Nur77 protein was purchased from Abcam (Cambridge, UK). Ubiquitin and all ubiquitin mutants were cloned into pCMV-HA-N vector (Clontech, Mountain View, CA).

### Cell culture and RNA interference

U2OS, HeLa, and 293FT cells were cultured in RPMI medium supplemented with 10% fetal bovine serum (FBS). All cell cultures followed standard procedures. Cultured cells were transfected using Lipofectamine 3000 reagents for plasmid transfections and LipoRNAiMax (Invitrogen; Thermo Fisher Scientific, Carlsbad, CA) for siRNA transfections. *Nur77* siRNA (5′-CAG CAU UAU GGU GUC CGC ACA UGU G-3′) and *Trim13* siRNA (5′-UAA UGA AUG UGCCAG UGU CUU GAG G-3′) were purchased from Invitrogen (Thermo Fisher Scientific). *CK2*α siRNA (sc-29918) was purchased from Santa-Cruz biotechnology.

### Antibodies and immunoblotting

The following antibodies were used in this study: a polyclonal anti-Nur77 antibody (Cell Signaling Technology, Beverly, MA or Proteintech, Rosemont, IL); an anti-Flag-M2 antibody (Sigma-Aldrich, St. Louis, MO); an anti-Myc antibody, an anti-HA antibody, and an anti-GFP antibody, anti-CK2α antibody (Santa Cruz Biotechnology, Dallas, TX); as well as the appropriate horseradish peroxidase-conjugated secondary antibodies (Cell Signaling Technology). Cell extracts were prepared in NP-40 buffer (50 mM Tris-HCl [pH 8.0], 105 mM NaCl, 1% NP-40, 1% SDS, and protease inhibitor cocktail [Roche Diagnostics, Indianapolis, IN]). Proteins were resolved by performing SDS-PAGE and were transferred onto an Amersham Protran NC membrane (GE Healthcare Life science, Freiburg, Germany). Immunoblots were visualized using the LAS-3000 system (GE Healthcare Life science). Densitometric analysis was carried out using LAS-3000 Image Reader and MultiGauge 3.0 software.

### Silver staining and LC-MS/MS

Protein separation was performed by 10% SDS-PAGE, the gels was fixed with 50% methanol/12% acetic acid containing 0.05% formalin at room temperature for 1 h, and washed with 35% ethanol. The gels were sensitized with 0.02% sodium thiosulfate for 2 min and then washed with water for 15 min. After incubation with 0.2% silver nitrate containing 0.076% formalin, the gels were developed in 6% sodium carbonate containing 0.0004% sodium thiosulfate and 0.05% formalin. The development reaction was stopped in 50% methanol and 12% acetic acid. Significant bands were cut, digested, and analyzed by LC-MS/MS from ProteomTech Inc (Daejeon, South Korea). In this sample, peptide match scores above 17 were considered statically significant.

### Generation of Nur77 phospho-specific antibody

Phospho-specific antibody against Ser154 site of Nur77 was generated in rabbits by Antibody Frontier (Seoul, South Korea) using the phospho-peptide QPSQL(pS)PWDGS. Antibodies against Ser154 site showed good responses and were affinity purified.

### Ubiquitination assay

Cells were transfected with vectors expressing each gene as indicated. At 36 h after transfection, the cells were treated with the proteasome inhibitor MG132 (10 μM) for 5 h. Next, the cells were collected in PBS and 5%~10% of the cell suspension was stored as input. The remainder of the cell suspension was centrifuged for 5 min, and the cells were then lysed in 700 μl of ubiquitination buffer (0.5% Nonidet P-40, 50 mM Tris-HCl [pH 7.4], 150 mM NaCl, 1 mM EDTA, 2 mM MgCl_2_, 5 mM *N*-ethylmaleimide, and protease inhibitor cocktail). The lysates were then centrifuged at 12,000 × g for 10 min, and then incubated with 1.5% SDS and 1 mM dithiothreitol at 65 °C for 20 min in order to denature the proteins, after which they were diluted 10-fold with lysis buffer. The denatured lysates were incubated overnight with the indicated antibodies and protein A/G PLUS agarose (Santa Cruz Biotechnology, Dallas, TX), resolved by SDS-PAGE, and immunoblotted with the indicated antibodies. For *in vitro* ubiquitination assay, recombinant Nur77 protein was purchased from Abcam (Cambridge, UK). Recombinant Trim13 protein was obtained from Flag-tagged Trim13 expressing HeLa cells and purified using anti-Flag-M2 affinity gel (Sigma-Aldrich, St. Louis, MO). The *in vitro* ubiquitination assay of Nur77 was performed using VIVAlink ubiquitin kit (VIVA bioscience, Exeter, UK). The reaction mixtures (ubiquitin assay buffer, 1 μM E1, 25 μM E2, 100 μM ubiquitin, 50 mM Mg-ATP, 100 ng recombinant Nur77 and Trim13 protein) were incubated at 37 °C for 2 h. Ubiquitinated Nur77 was detected by western blotting using an ubiquitin antibody.

### Protein half-life analysis

A cycloheximide (CHX) blocking analysis was performed to determine the half-life of Nur77. Cells were incubated with CHX (50 μg/ml) for various times, and Nur77 was detected by western blot analysis. The density of the immunoreactive bands corresponding to Nur77 and β-actin were measured. The level of Nur77 was quantified by normalization with β-actin, and the percentage of remaining Nur77 was plotted.

### Quantification of IL-6 production by EIA

The cell culture supernatants were collected at various time points treated or untreated with the indicated ligand or inhibitors. The protein standards and antibody pairs for IL-6 was procured from R&D systems (Minneapolis, MN). EIA experiment for IL-6 measurement was performed according to the manufacturer’s instructions.

### Co-immunoprecipitation

For the co-immunoprecipitation assay, cells grown in 10 cm dishes were transfected with various plasmids, and lysates were prepared 36 h after transfection. Cells were lysed in RIPA buffer (20 mM HEPES [pH 7.5], 1% NP-40, 0.1% SDS, 0.5% deoxycholic acid, and 150 mM NaCl) containing 10 μM NaF and protease inhibitor cocktail. Cell lysates were incubated with the appropriate antibody for overnight and subsequently incubated with protein A-Sepharose beads for 1 h. The protein-antibody complexes recovered on beads were subjected to western blotting using appropriate antibodies after separation by SDS-PAGE.

### Purification of Trim13 recombinant protein

HeLa cells were grown in 10 cm dishes and were transfected with Flag vector or Flag-Trim13 plasmids (each ten dishes). After 36 h, the cells were washed two times in ice-cold PBS, and lysed in cell lysis buffer containing protease inhibitors. Flag-Trim13 lysate was incubated with Flag beads overnight. The beads were extensively washed with the lysis buffer containing protease inhibitors. The protein bound to beads was eluted with the Flag peptide and the beads were centrifuged and the supernatant containing Trim13 proteins were stored at −80 °C before CBB staining and *in vitro* ubiquitination assays were performed.

### Statistical analysis

Optical intensity was measured using the AlphaEase program (Version 5.1; Alpha Innotech, San Leandro, CA). Data were analyzed using GraphPad Prism Version 4 (GraphPad Software Inc., San Diego, CA). All numerical values are presented as mean ± SD. Statistical significance was determined using an unpaired Student’s *t*-test.

## Electronic supplementary material

Supplementary information
