# Supplementary material for: The E3 ubiquitin ligase Trim13 regulates Nur77 stability via casein kinase 2α
Source: Sci Rep. 2018 Sep 17;8:13895. doi: 10.1038/s41598-018-32391-5 (PMC6141542; doi:10.1038/s41598-018-32391-5)

Supplementary information

The E3 ubiquitin ligase Trim13 regulates Nur77 stability *via* casein kinase 2α

Bin Huang, Han Zhong Pei, Hyeun-Wook Chang and Suk-Hwan Baek

Uncropped scans of blots and gels displayed in the main figures.

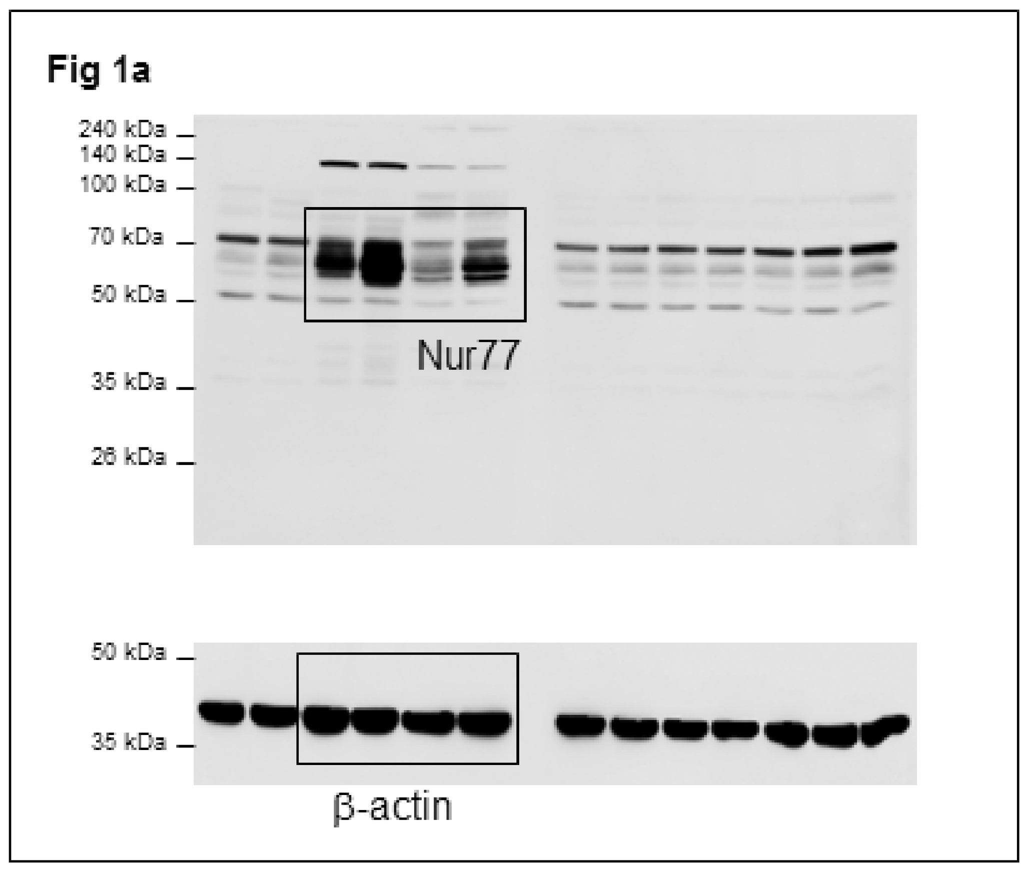
Fig 1a
240 kDa
140 kDa
100 kDa
70 kDa
50 kDa
35 kDa
26 kDa
Nur77
50 kDa
35 kDa
β-actin

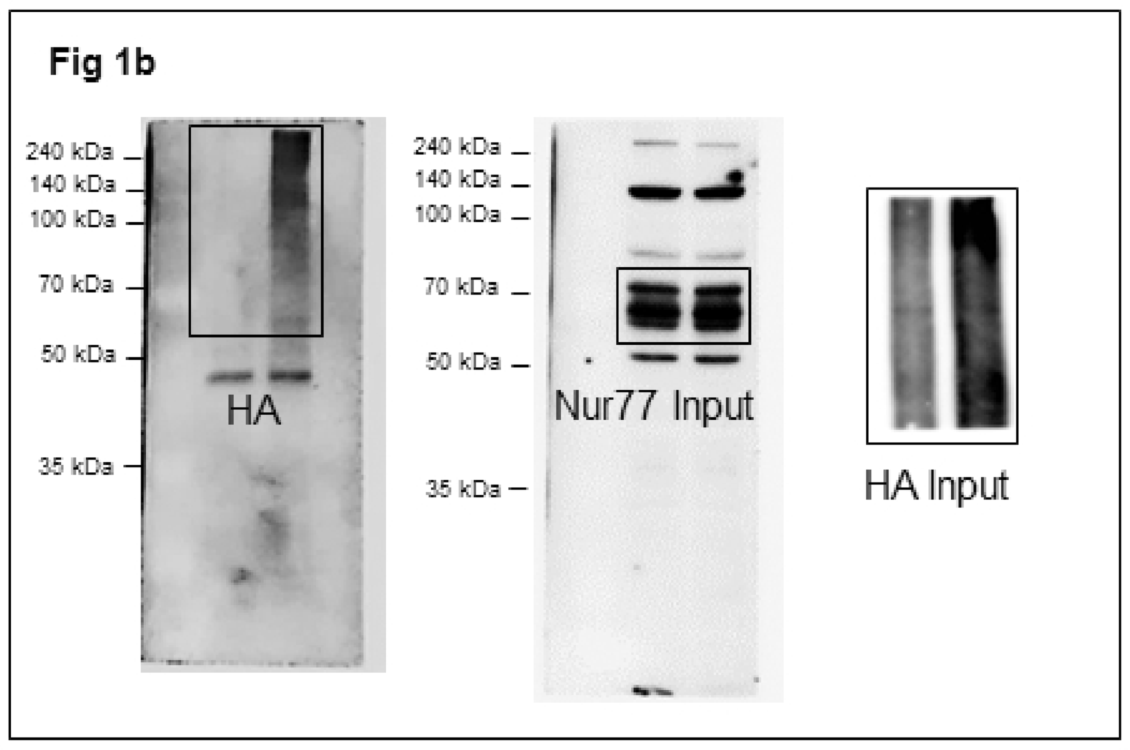
Fig 1b
240 kDa
140 kDa
100 kDa
70 kDa
50 kDa
35 kDa
HA
Nur77 Input
HA Input

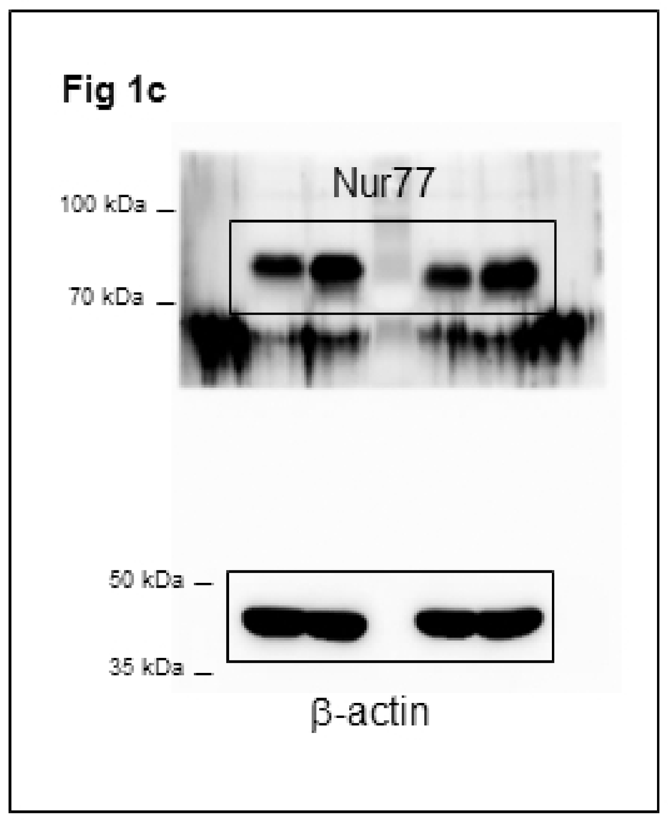
Fig 1c
100 kDa
70 kDa
Nur77
50 kDa
35 kDa
β-actin

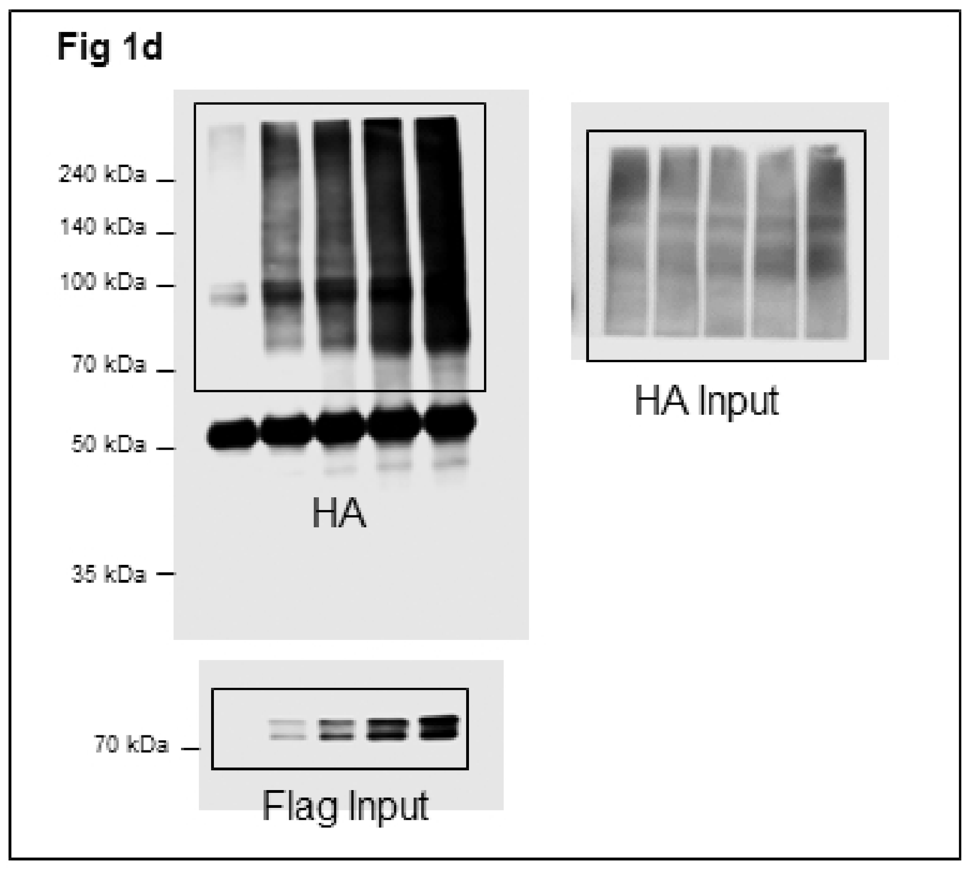
Fig 1d
240 kDa
140 kDa
100 kDa
70 kDa
50 kDa
35 kDa
HA
HA Input
70 kDa
Flag Input

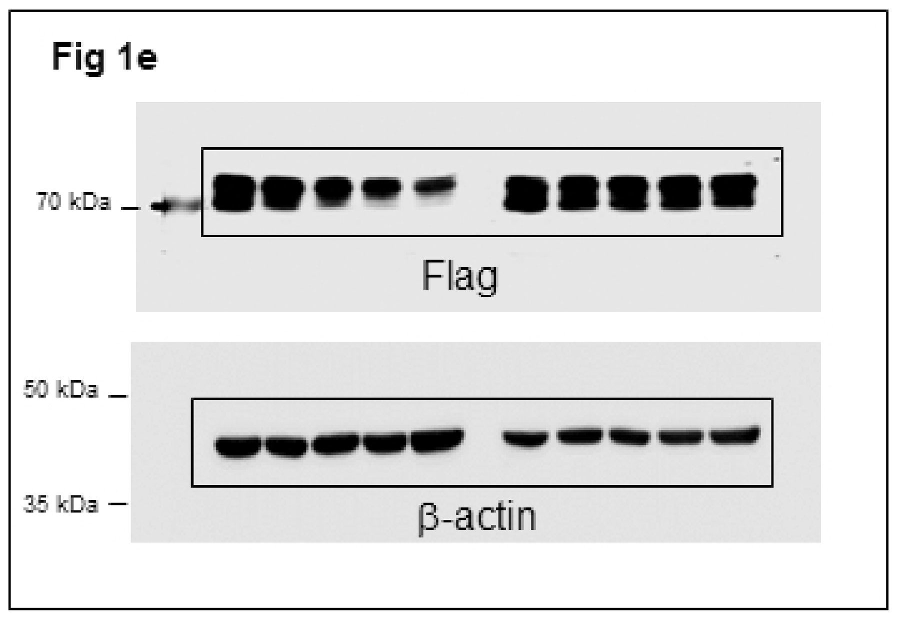
Fig 1e
70 kDa
Flag
50 kDa
35 kDa
β-actin

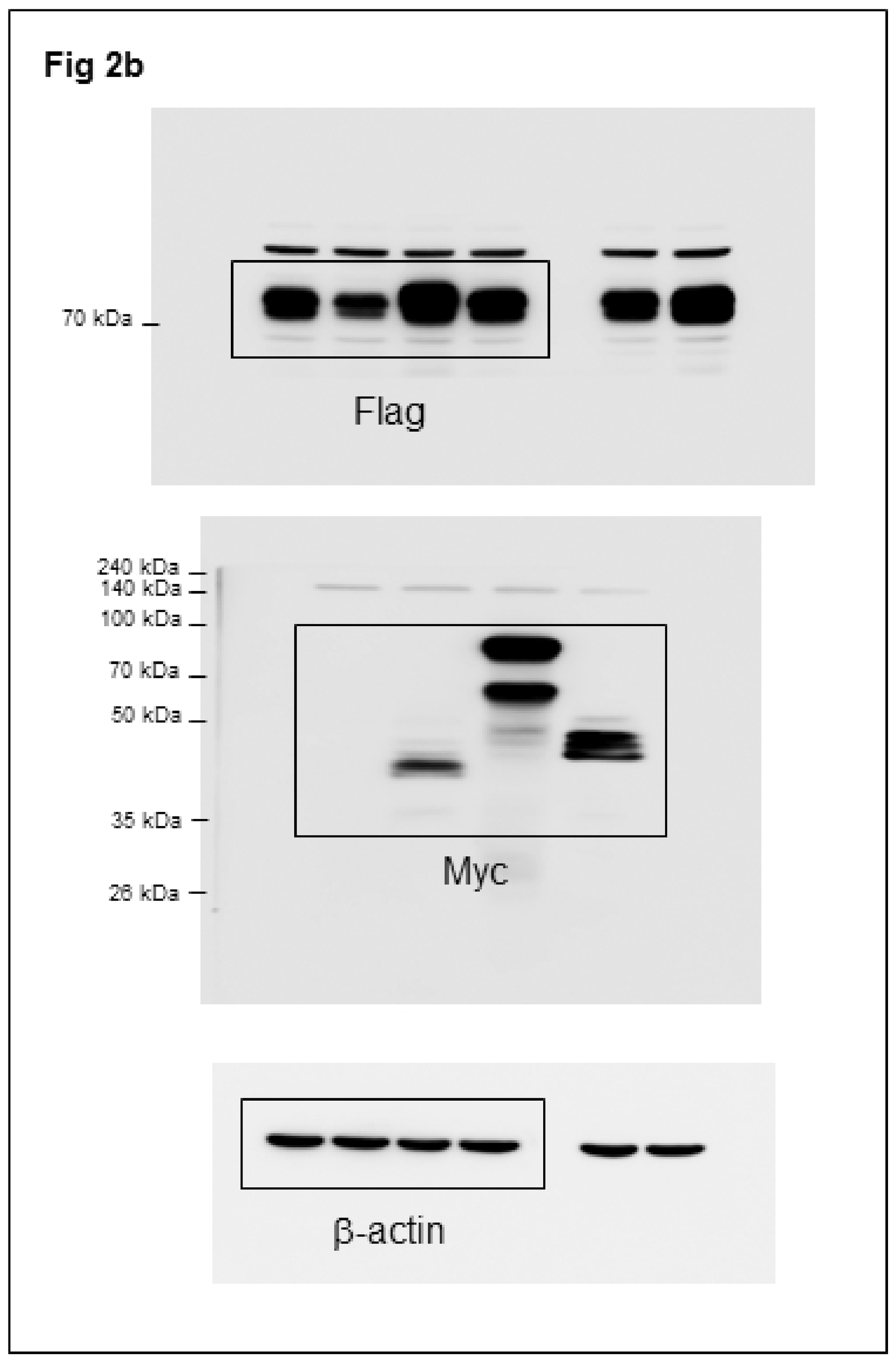
Fig 2b
70 kDa
Flag
240 kDa
140 kDa
100 kDa
70 kDa
50 kDa
35 kDa
26 kDa
Myc
β-actin

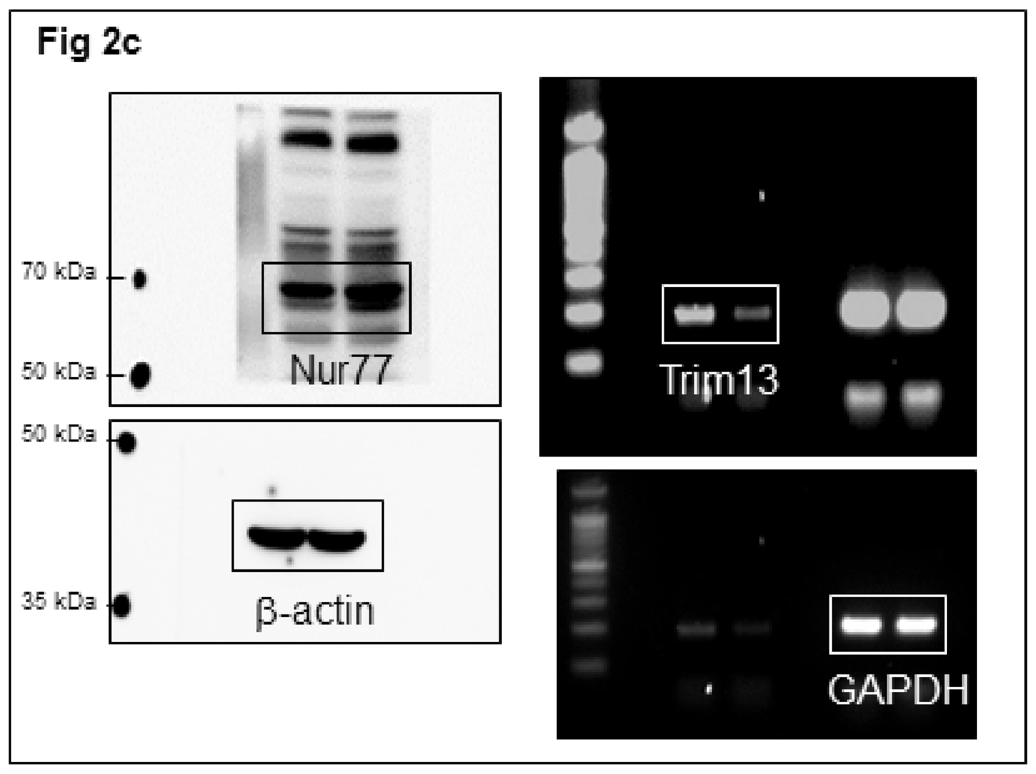
Fig 2c
70 kDa
50 kDa
Nur77
Trim13
50 kDa
35 kDa
β-actin
GAPDH

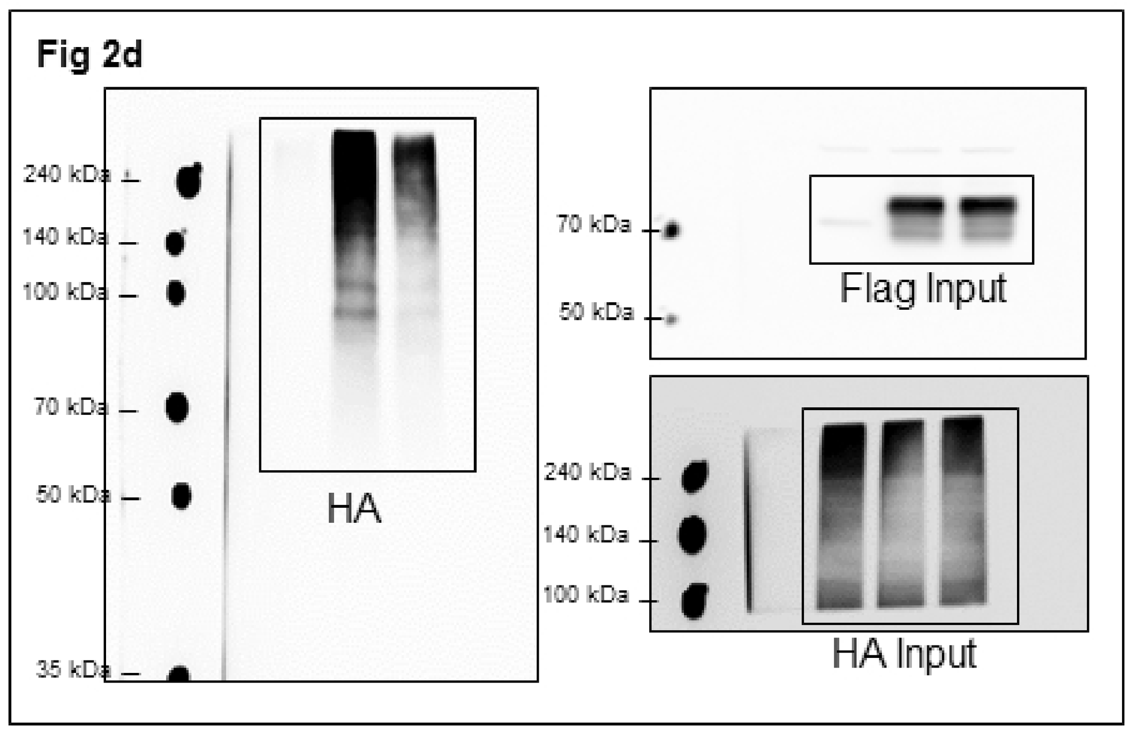
Fig 2d
240 kDa
140 kDa
100 kDa
70 kDa
50 kDa
35 kDa
HA
70 kDa
50 kDa
Flag Input
240 kDa
140 kDa
100 kDa
HA Input

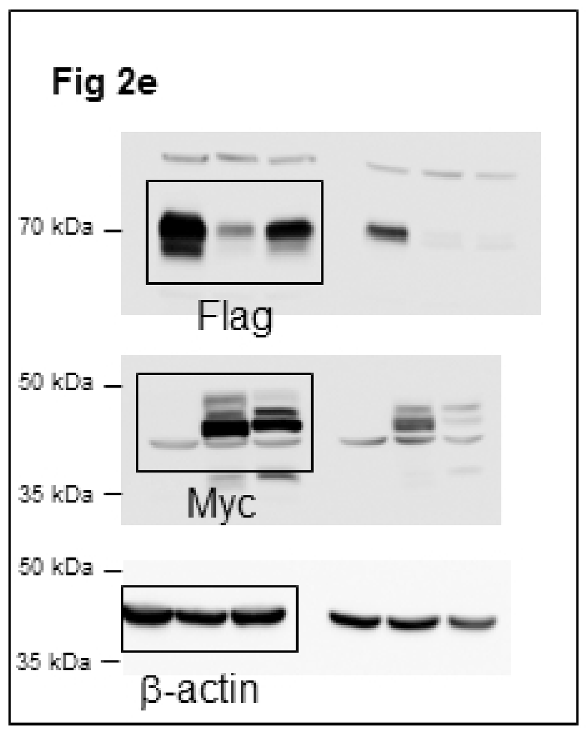
Fig 2e
70 kDa
Flag
50 kDa
35 kDa
Myc
50 kDa
35 kDa
β-actin

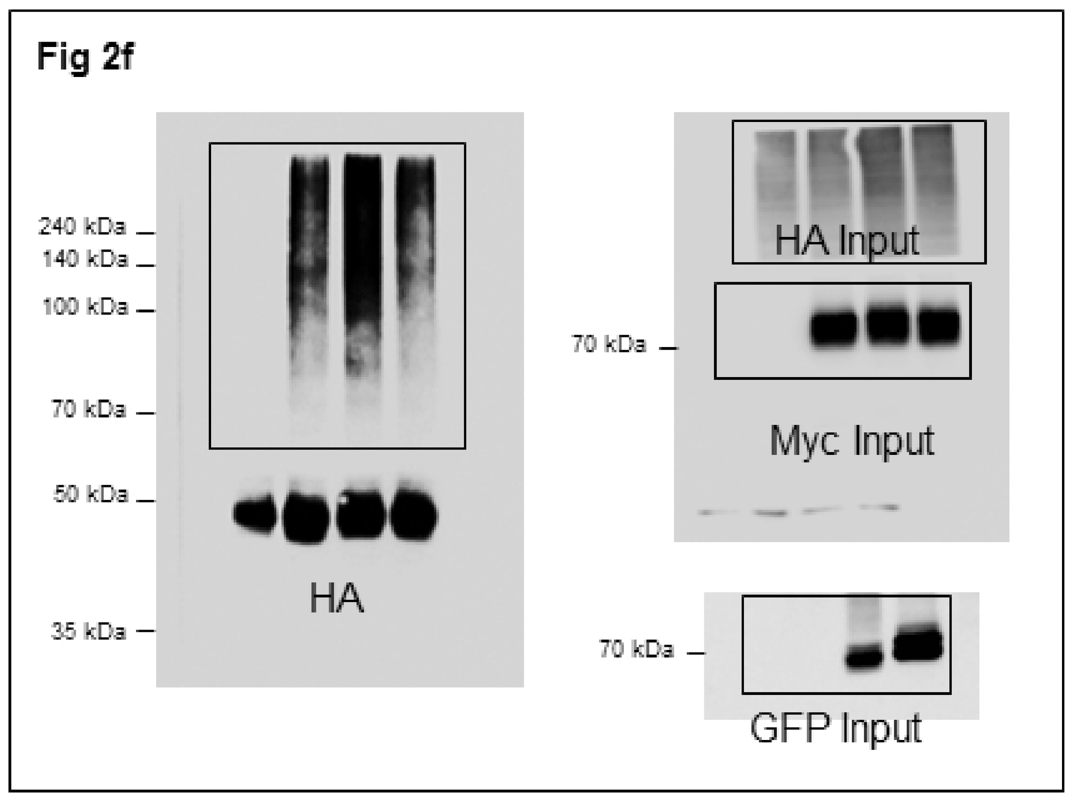
Fig 2f
240 kDa
140 kDa
100 kDa
70 kDa
50 kDa
35 kDa
HA
HA Input
70 kDa
Myc Input
70 kDa
GFP Input

Fig 2g

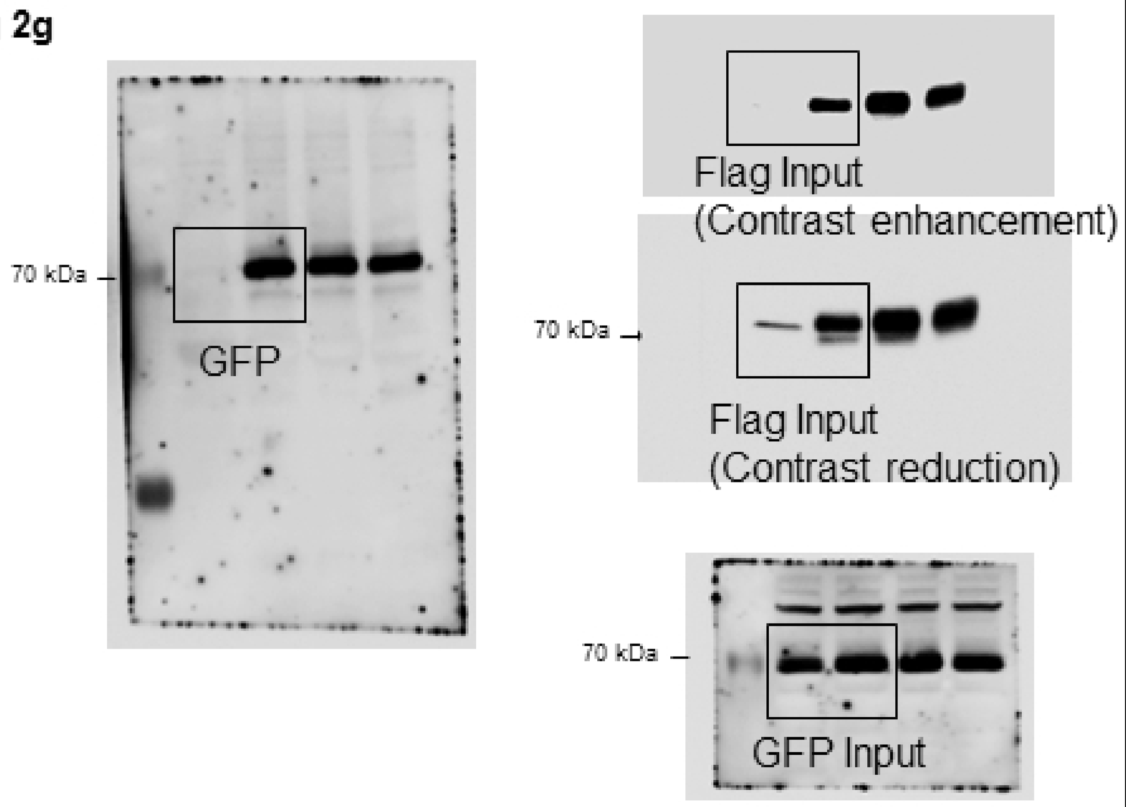

Fig 2h

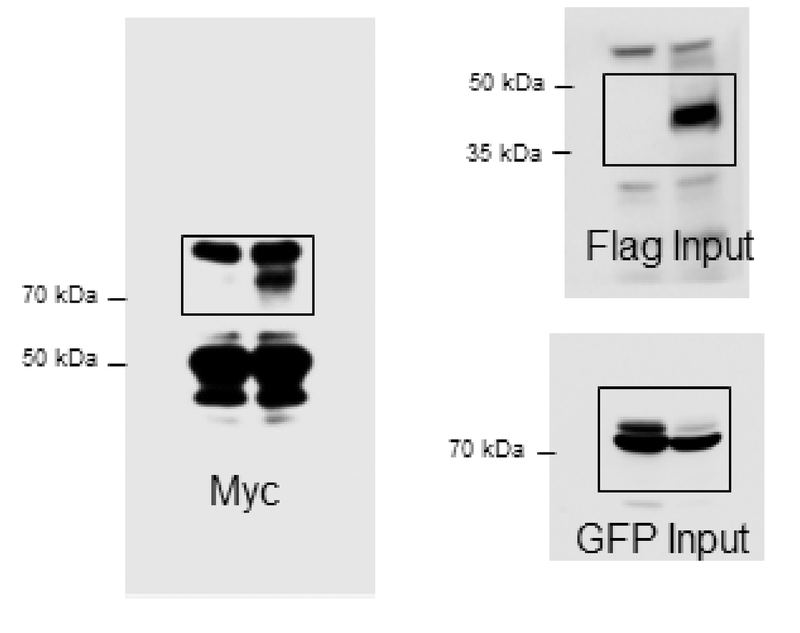

Fig 3a

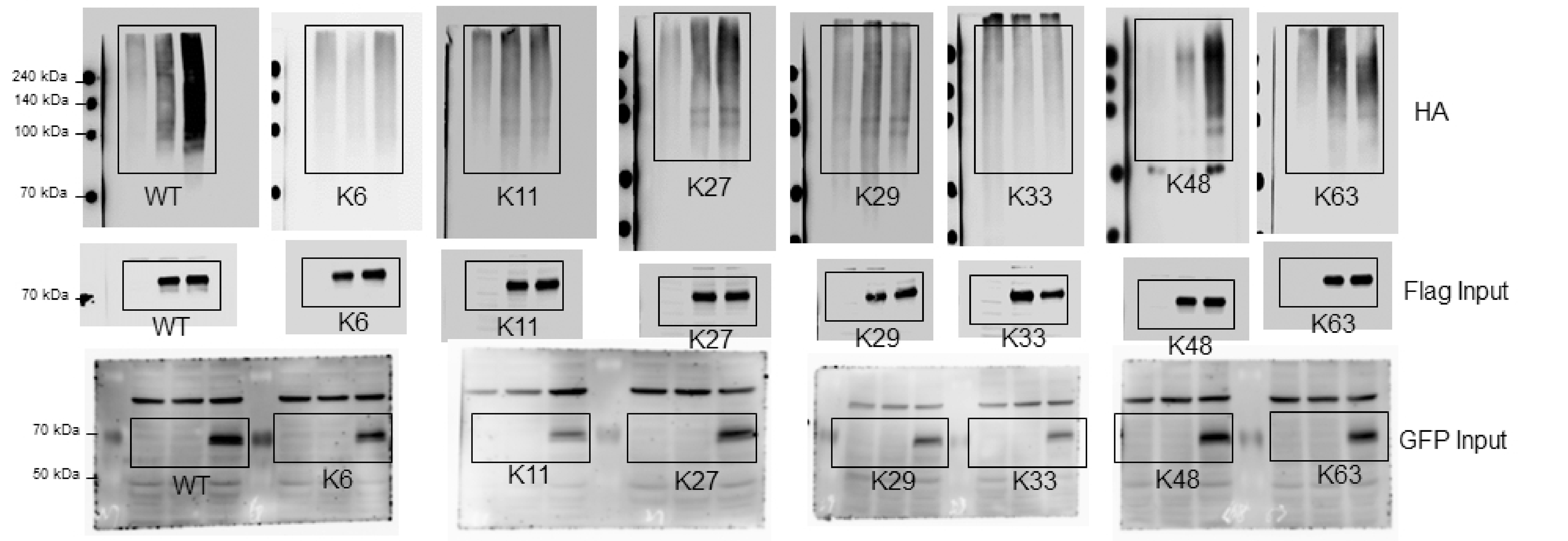

Fig 3b

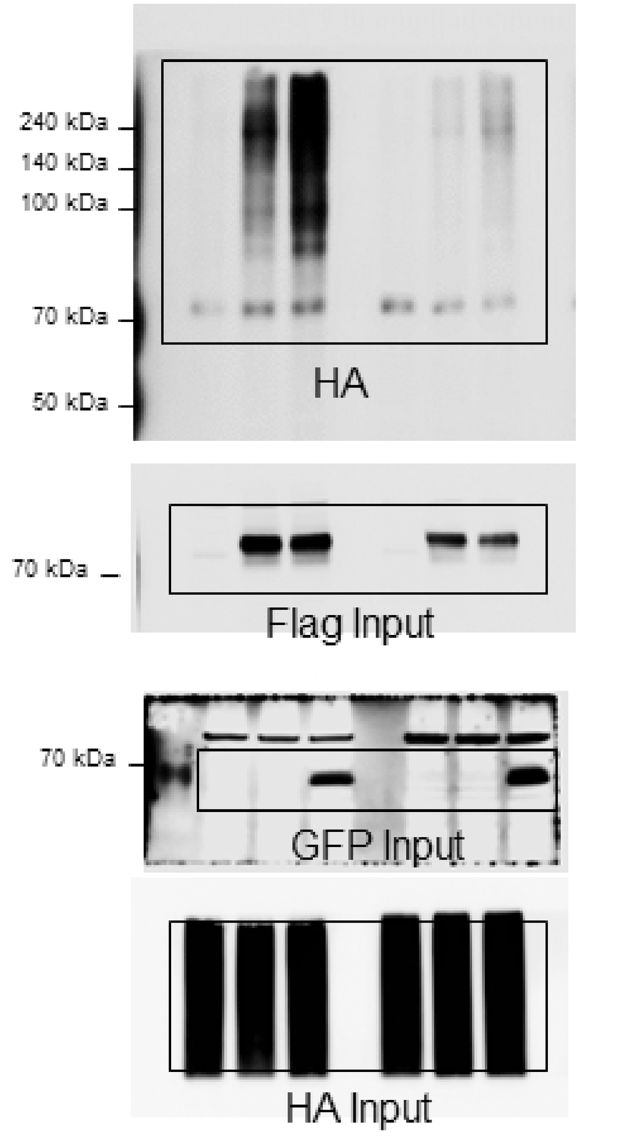

Fig 3c

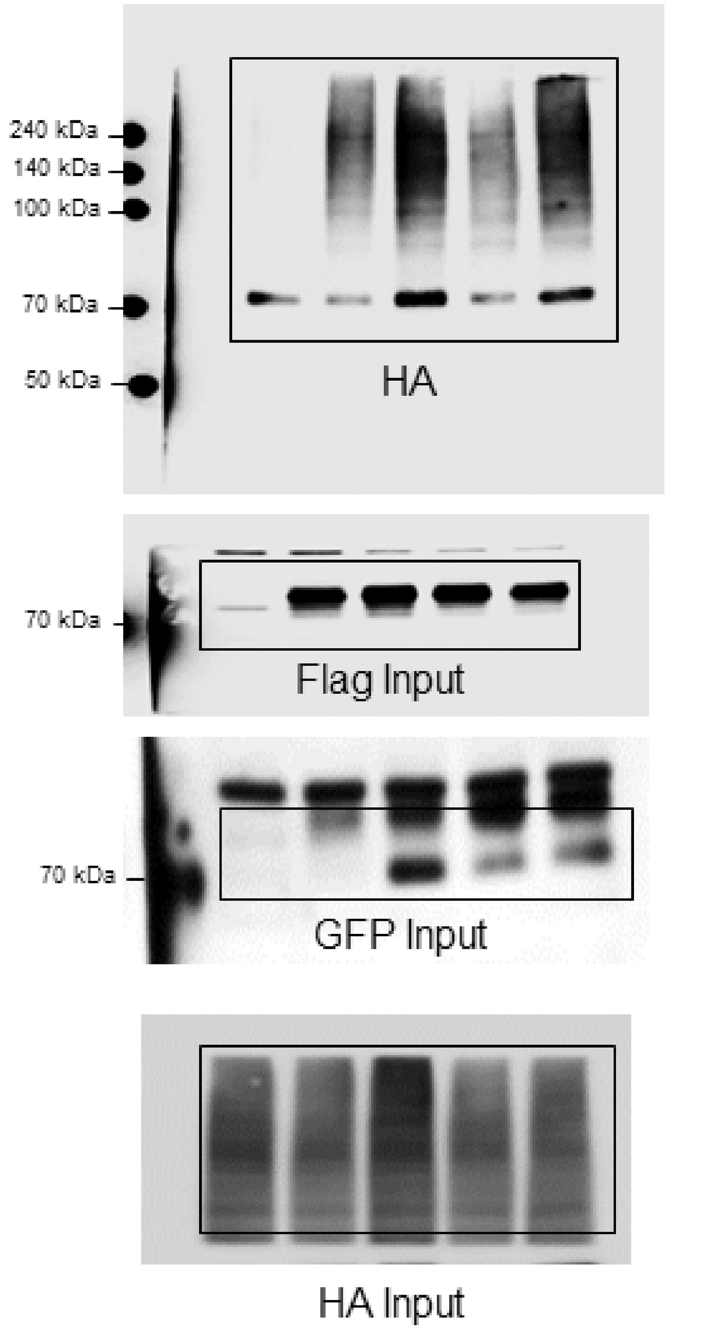

Fig 4a

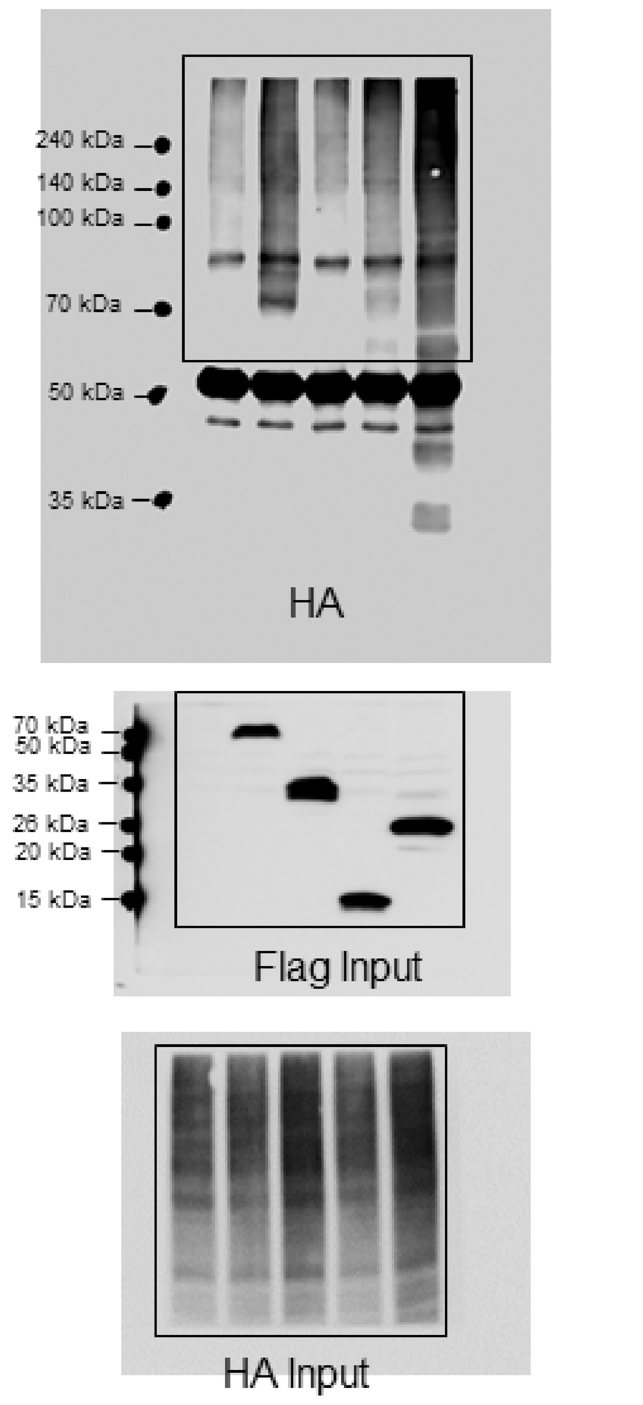

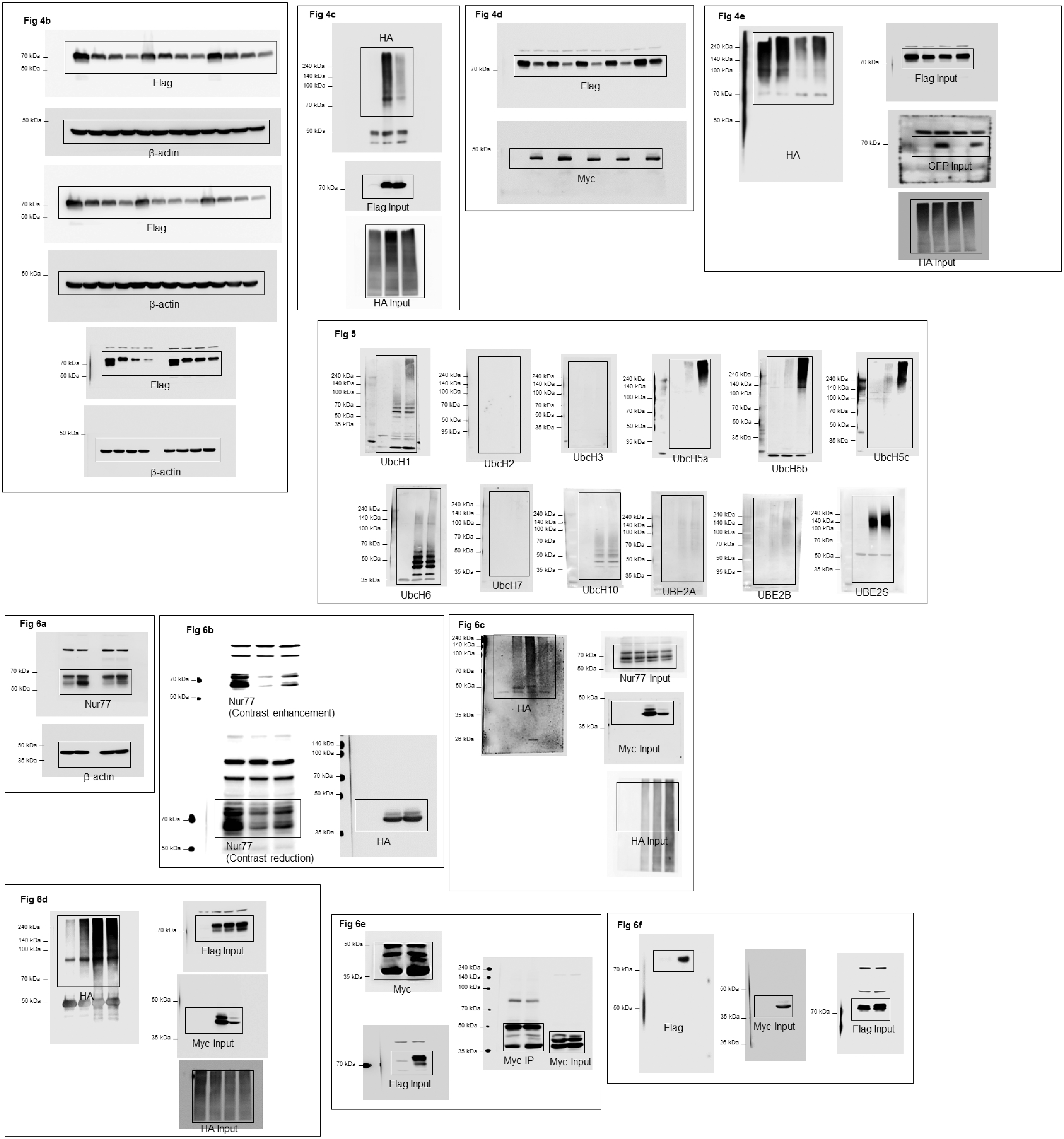

Fig 4b
Flag
β-actin
Flag
β-actin
Flag
β-actin
Fig 4c
HA
Flag Input
HA Input
Fig 4d
Flag
Myc
Fig 4e
HA
Flag Input
GFP Input
HA Input
Fig 5
UbcH1
UbcH2
UbcH3
UbcH5a
UbcH5b
UbcH5c
UbcH6
UbcH7
UbcH10
UBE2A
UBE2B
UBE2S
Fig 6a
Nur77
β-actin
Fig 6b
Nur77
(Contrast enhancement)
Nur77
(Contrast reduction)
HA
Fig 6c
HA
Nur77 Input
Myc Input
HA Input
Fig 6d
HA
Flag Input
Myc Input
HA Input
Fig 6e
Myc
Flag Input
Myc IP
Myc Input
Fig 6f
Flag
Myc Input
Flag Input

Fig 7b

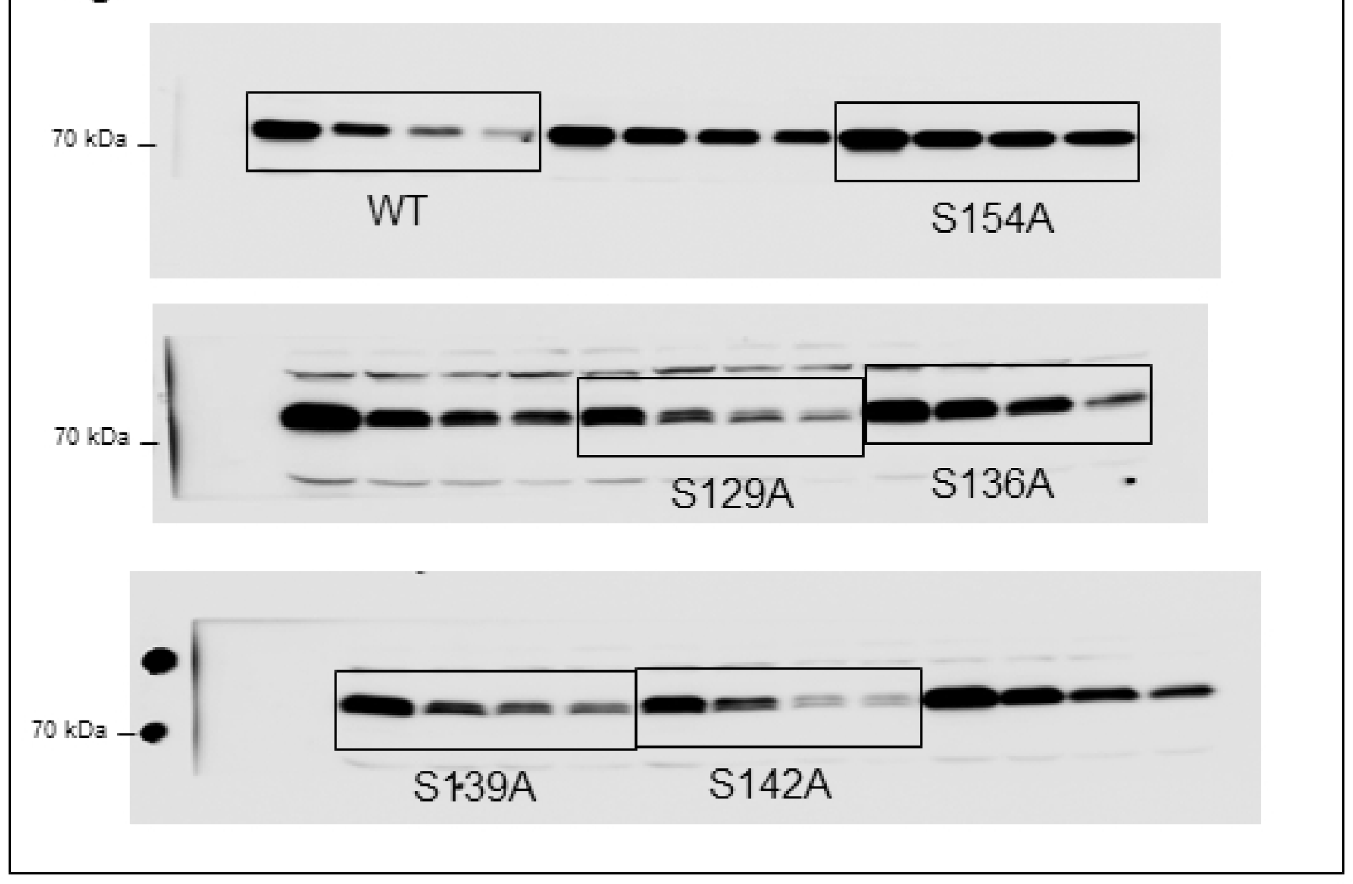

Fig 7c

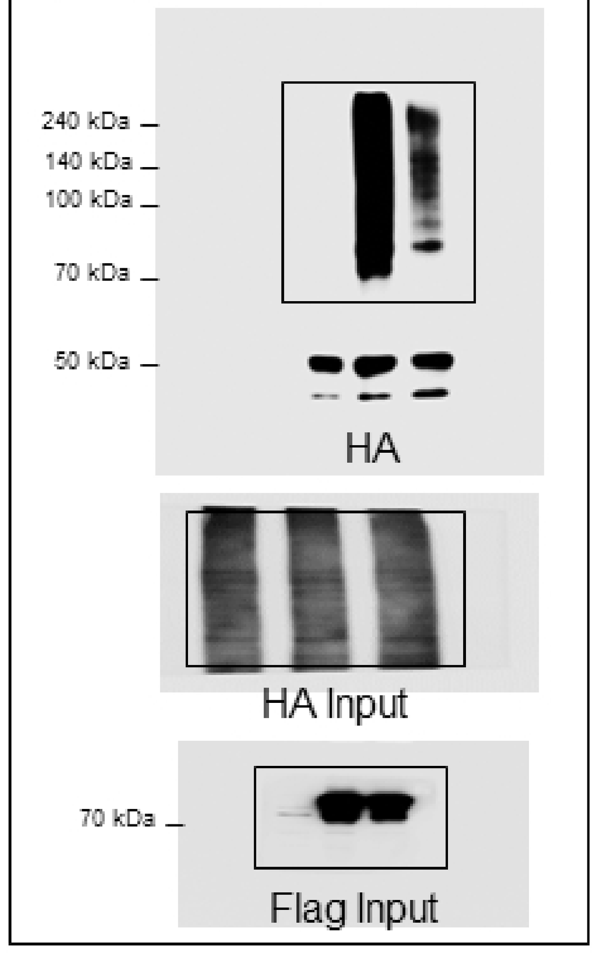

Fig 7d

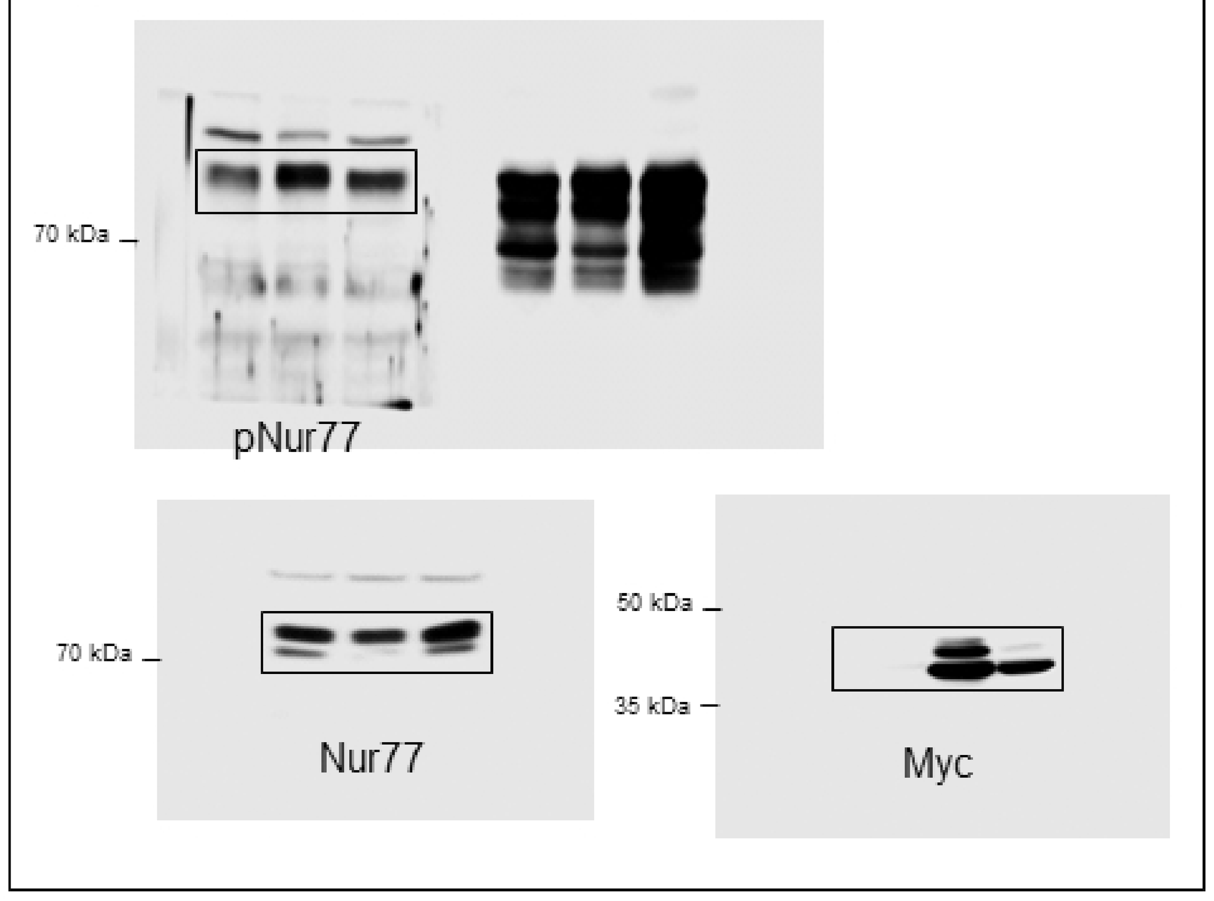

Fig 7e

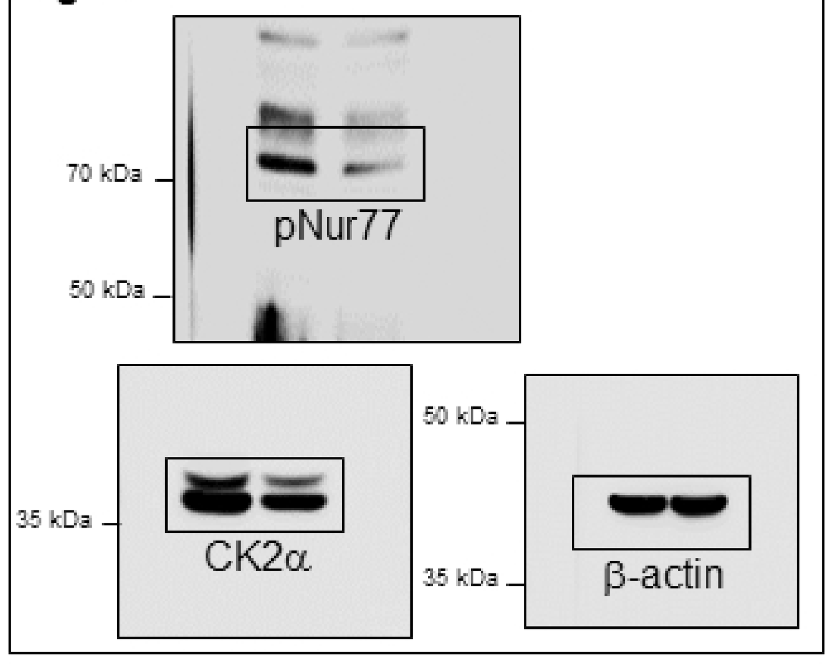

Fig 7f

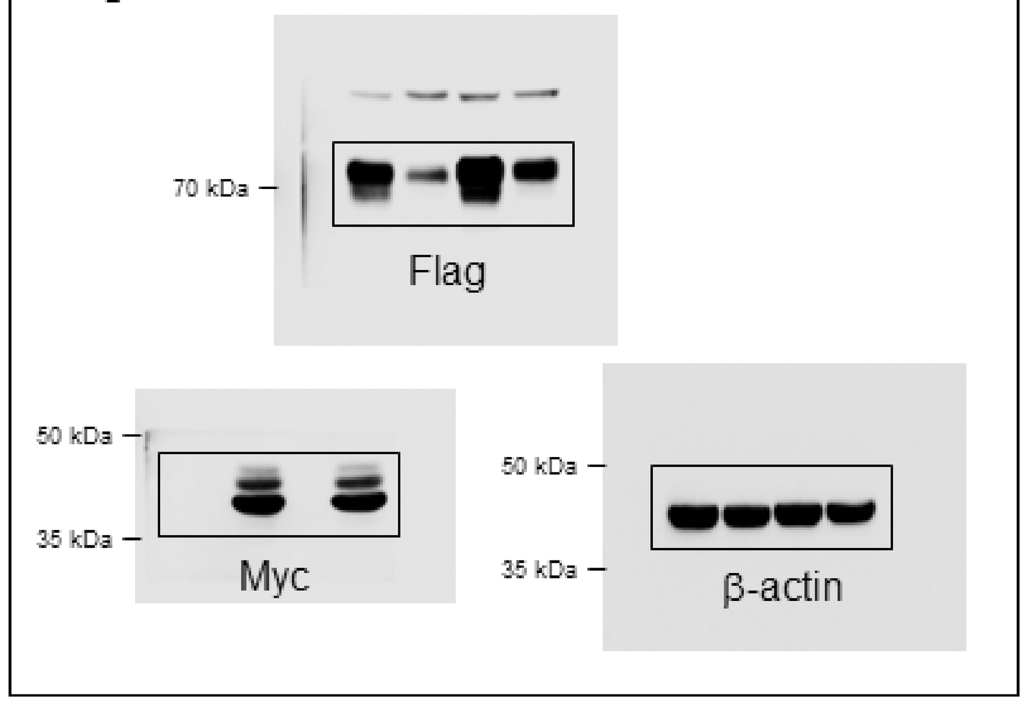

Fig 7g

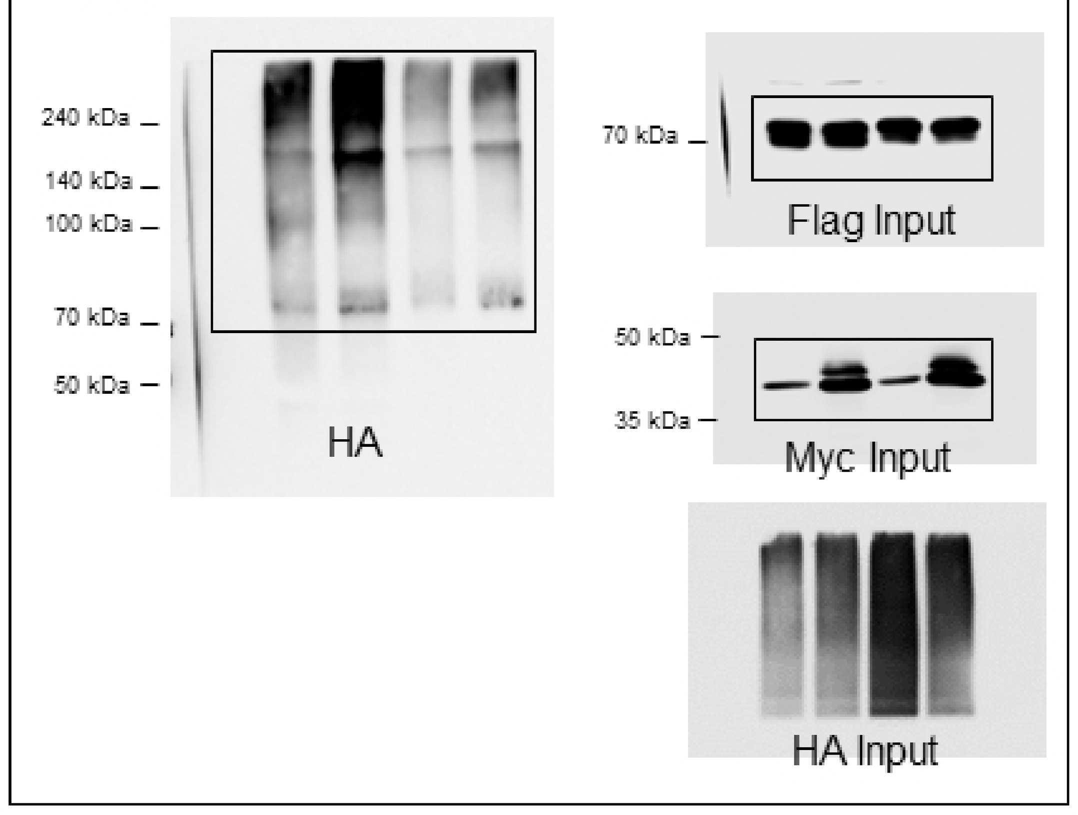

Fig 8a

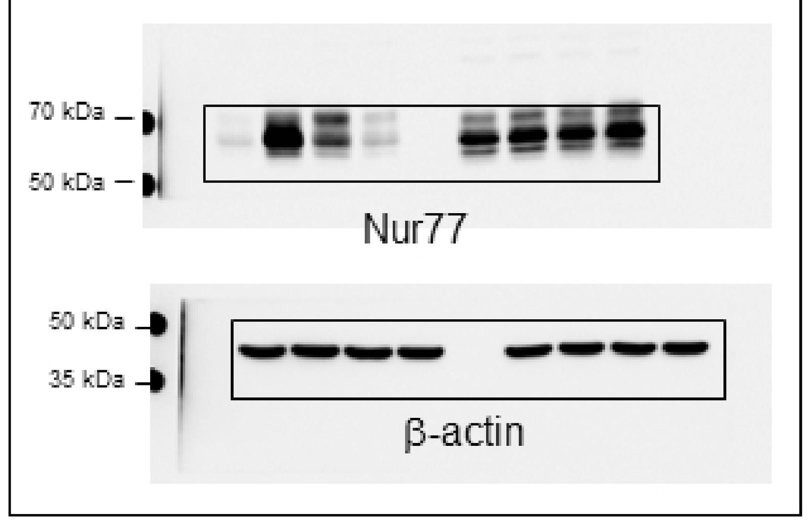

Fig 8b

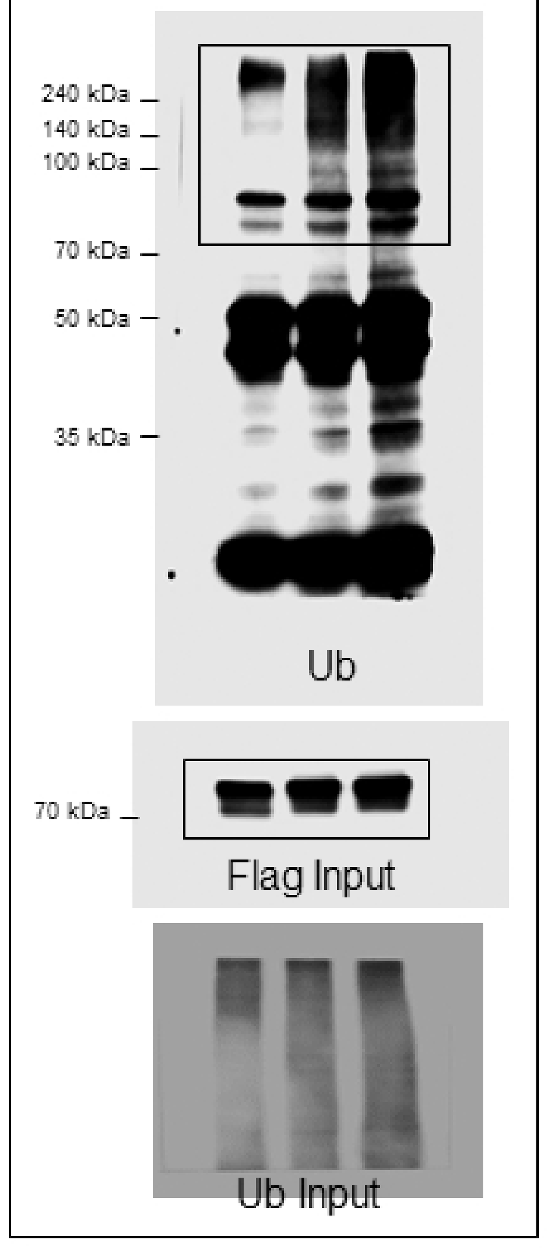

Fig 8c

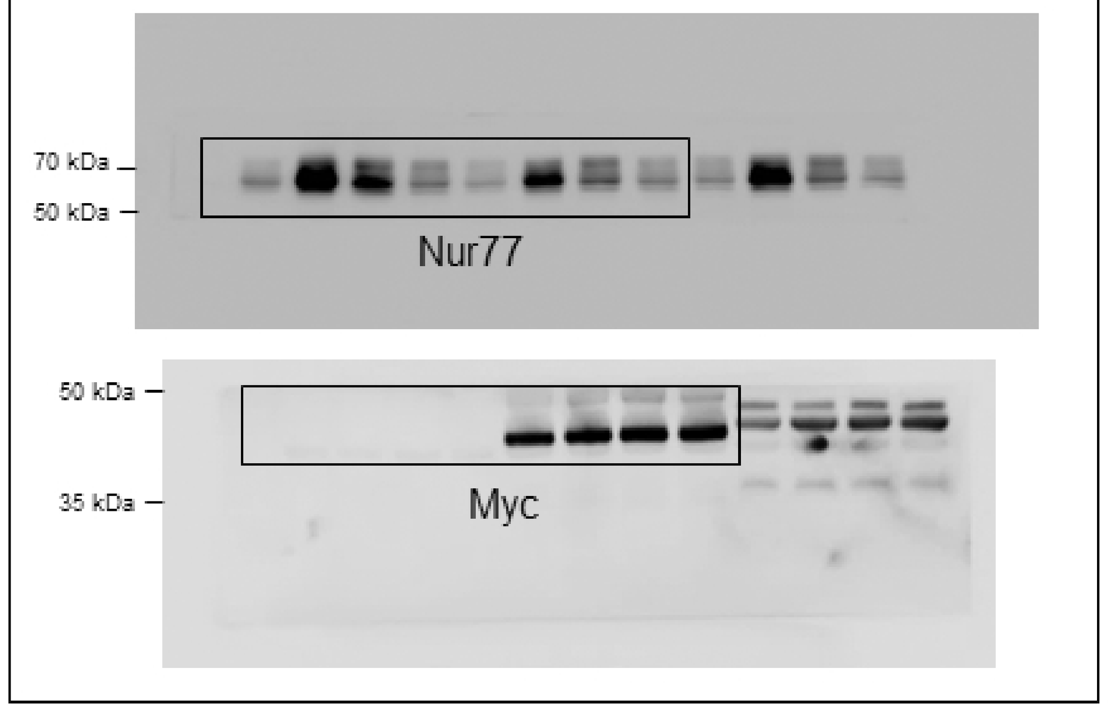

Fig 8d

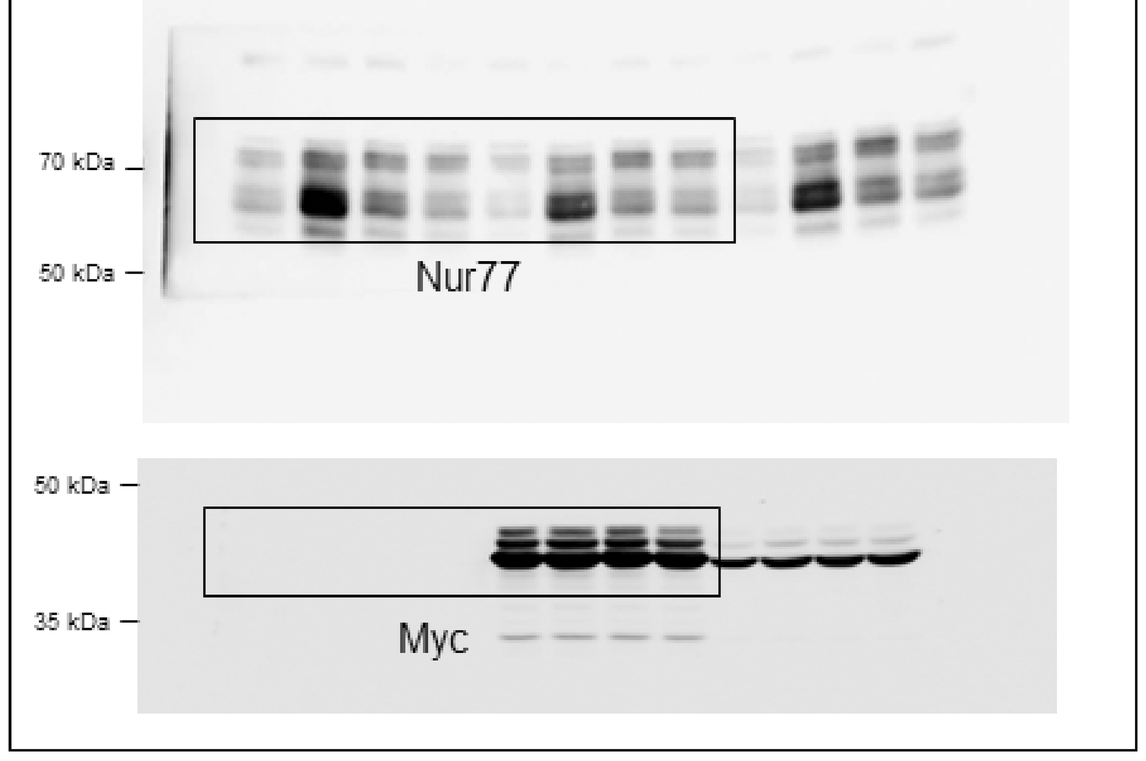

Fig 8e

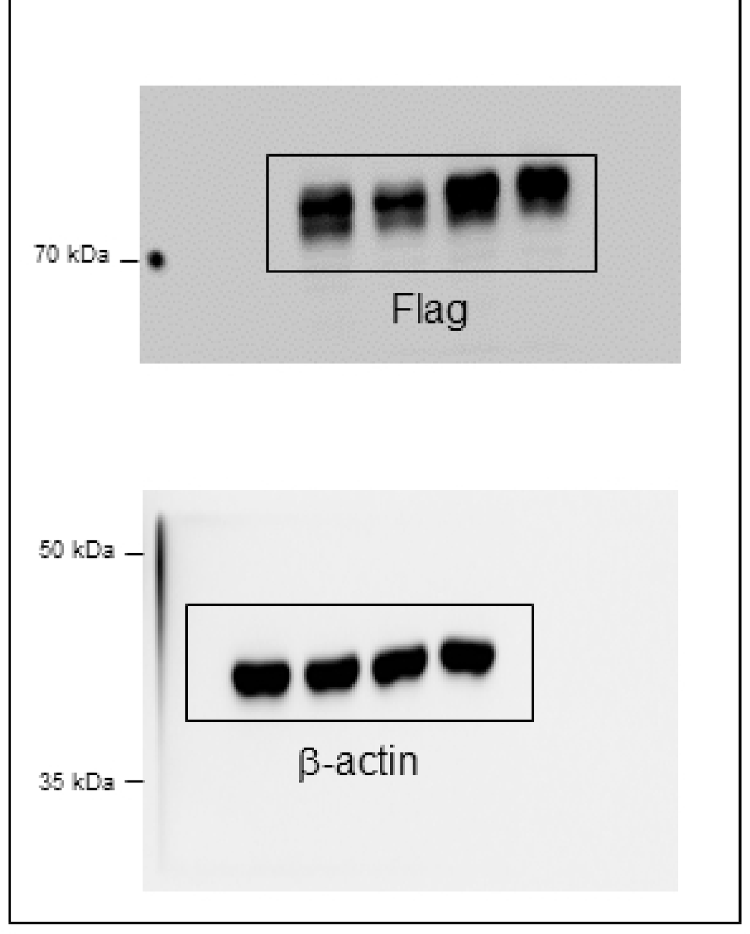

Fig 8f

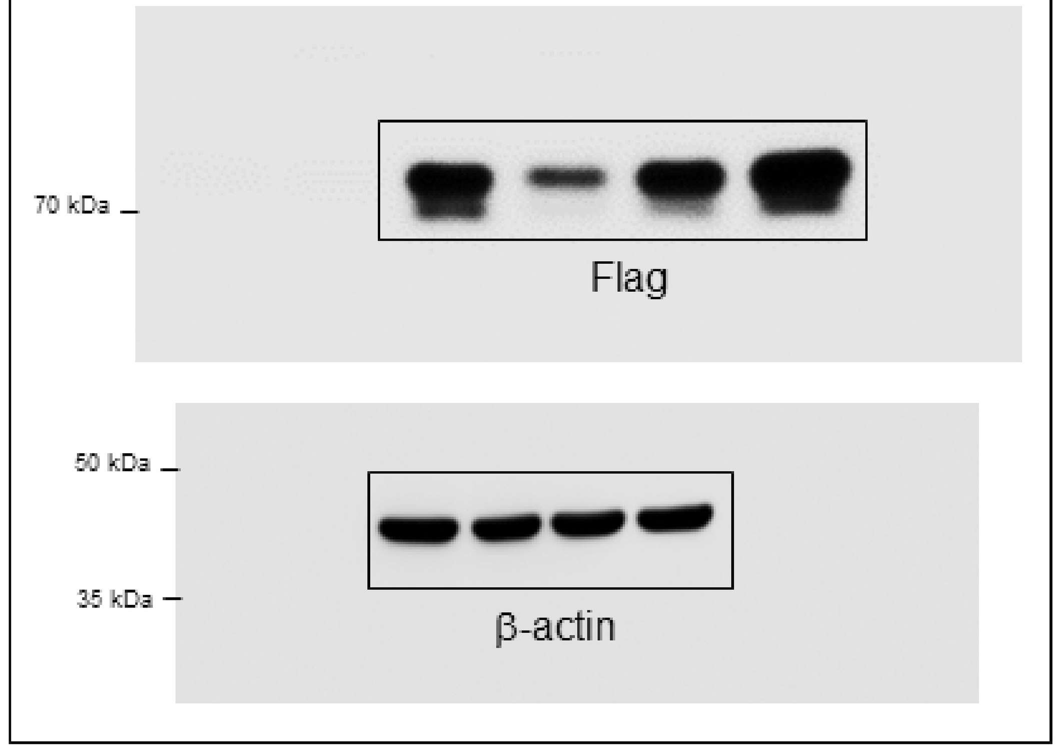

Fig 9a

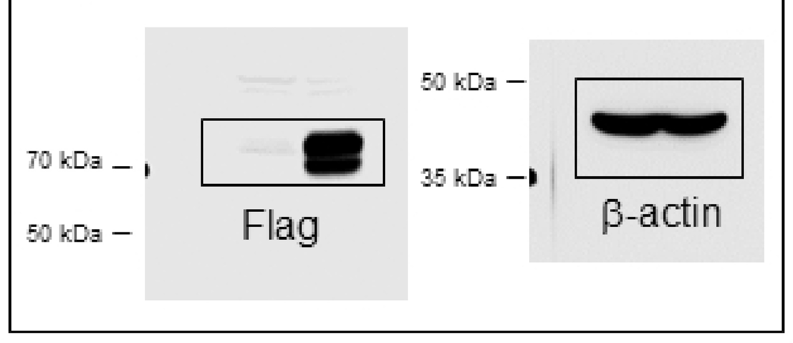

Fig 9b

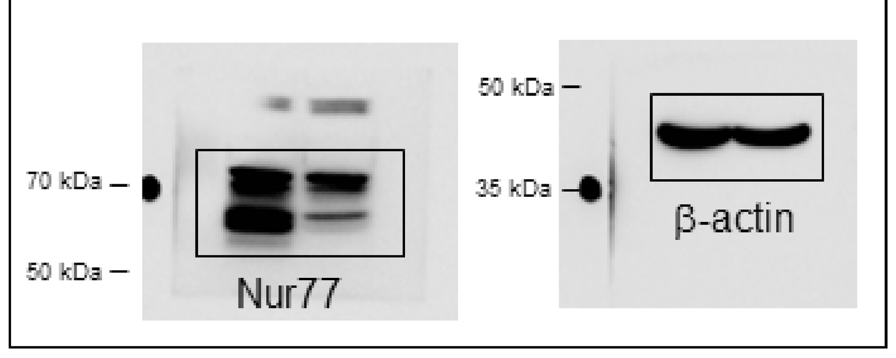

Supplement: Supplementary file 1 — Supplementary information [file 41598_2018_32391_MOESM1_ESM.pdf]
